# Phylogeny of Campanuloideae (Campanulaceae) with Emphasis on the Utility of Nuclear Pentatricopeptide Repeat (PPR) Genes

**DOI:** 10.1371/journal.pone.0094199

**Published:** 2014-04-09

**Authors:** Andrew A. Crowl, Evgeny Mavrodiev, Guilhem Mansion, Rosemarie Haberle, Annalaura Pistarino, Georgia Kamari, Dimitrios Phitos, Thomas Borsch, Nico Cellinese

**Affiliations:** 1 Florida Museum of Natural History, University of Florida, Gainesville, Florida, United States of America; 2 Department of Biology, University of Florida, Gainesville, Florida, United States of America; 3 Botanischer Garten und Botanisches Museum, Freie Universität Berlin, Berlin, Germany; 4 Biology Department, Pacific Lutheran University, Tacoma, Washington, United States of America; 5 Museo Regionale di Scienze Naturali, Torino, Italy; 6 Department of Biology, University of Patras, Patras, Greece; University of Leicester, United Kingdom

## Abstract

**Background:**

The Campanuloideae (Campanulaceae) are a highly diverse clade of angiosperms found mostly in the Northern Hemisphere, with the highest diversity in temperate areas of the Old World. Chloroplast markers have greatly improved our understanding of this clade but many relationships remain unclear primarily due to low levels of molecular evolution and recent and rapid divergence. Furthermore, focusing solely on maternally inherited markers such as those from the chloroplast genome may obscure processes such as hybridization. In this study we explore the phylogenetic utility of two low-copy nuclear loci from the pentatricopeptide repeat gene family (PPR). Rapidly evolving nuclear loci may provide increased phylogenetic resolution in clades containing recently diverged or closely related taxa. We present results based on both chloroplast and low-copy nuclear loci and discuss the utility of such markers to resolve evolutionary relationships and infer hybridization events within the Campanuloideae clade.

**Results:**

The inclusion of low-copy nuclear genes into the analyses provides increased phylogenetic resolution in two species-rich clades containing recently diverged taxa. We also obtain support for the placement of two early diverging lineages (*Jasione* and *Musschia*-*Gadellia* clades) that have previously been unresolved. Furthermore, phylogenetic analyses of PPR loci revealed potential hybridization events for a number of taxa (e.g., *Campanula pelviformis* and *Legousia* species). These loci offer greater overall topological support than obtained with plastid DNA alone.

**Conclusion:**

This study represents the first inclusion of low-copy nuclear genes for phylogenetic reconstruction in Campanuloideae. The two PPR loci were easy to sequence, required no cloning, and the sequence alignments were straightforward across the entire Campanuloideae clade. Although potentially complicated by incomplete lineage sorting, these markers proved useful for understanding the processes of reticulate evolution and resolving relationships at a wide range of phylogenetic levels. Our results stress the importance of including multiple, independent loci in phylogenetic analyses.

## Introduction

Campanulaceae Jussieu are a nearly cosmopolitan group of flowering plants comprising five subfamilies (Campanuloideae, Lobelioideae, Nemacladoideae, Cyphioideae, and Cyphocarpoideae), approximately 84 traditionally circumscribed genera, and more than 2300 species [1]. Historically, there has been much disagreement as to intrafamilial classification [2], [3], [4], primarily due to the polyphyly of the largest genera, *Campanula* and *Wahlenbergia* [5], [6], [7], [8], [9].

The heterogeneous Campanuloideae Burnett (approximately 1054 species) are found primarily in the Northern Hemisphere and are most abundant in temperate areas of the Old World [1], with major centers of diversity in the Mediterranean Basin and the Middle East. They are found from temperate to sub-tropical areas and occupy a wide variety of habitats, from steppes to high elevation mountainous regions. Some species have wide distribution ranges, spanning entire continents, while others are narrow endemics, e.g., restricted to single islands.

Previous studies of the Campanulaceae and Campanuloideae have typically focused on a few chloroplast genes [5], [6], [10], [11], one chloroplast marker with expanded taxon sampling [8], [12], or gene order in the highly rearranged chloroplast genome [13], [14], [15]. These studies have made significant progress toward a robust phylogenetic hypothesis of the group and have highlighted the high level of paraphyly and polyphyly of many traditionally circumscribed genera, especially *Campanula* and *Wahlenbergia*. However, species-level relationships are yet to be understood as the most widely used plastid markers within the family (i.e., *atpB*, *matK*, and *rbcL*) have not been able to provide a significant level of resolution. Furthermore, focusing solely on maternally inherited markers may obscure the role that hybridization may have played in the evolutionary history of this group.

Additional studies have attempted to use nuclear data by including the internal transcribed spacers (ITS) sequences of nuclear ribosomal DNA [7], [9], [11], [16], [17], [18], [19]. Although potentially informative at the species level, this region is considerably difficult to align with confidence in positional homology across wide phylogenetic distances in the Campanuloideae and is further complicated by potential concerted evolution and high levels of homoplasy (for further discussion and concerns see [20], but see [21]). Ultimately, past studies including ITS have shown its significant limitation in resolving species level relationships and providing accurate information on the placement of several genera (e.g., *Jasione* and *Musschia*).

Inferring robust phylogenies for species-rich clades is of great importance for understanding processes of speciation, hybridization, and patterns in historical biogeography while posing a major challenge to systematists [8], [12], [22]. Resolving relationships at low taxonomic levels can be difficult for taxa that are closely related and/or recently diverged. Furthermore, relationships at the interspecific level can be complicated by hybridization and introgression. Thus, multiple rapidly evolving, independent nuclear markers may be useful, and even necessary, to accurately reconstruct species-level phylogenies [23].

Current molecular and phylogenetic methods allow researchers to obtain large, multi-gene datasets for phylogenetic studies. However, because of highly conserved genome organization, gene order, and gene content of the chloroplast genome across much of angiosperm diversity (but see [13], [14], [15], [24] for exceptions in the Campanuloideae) and the relative ease of developing universal primers for both chloroplast and nuclear ribosomal DNA, these have been the most widely used sources of molecular data for plant phylogenetics [25]. Although universal markers are more labor-intensive to develop due to gene duplications and deletions [23], under-utilized low-copy nuclear genes can be of great value to molecular phylogenetic studies.

Low-copy nuclear genes have a number of advantages over chloroplast markers: they are unlinked [26], possess increased sequence variation [27], and are bi-parentally inherited [23], [25], [28]. Unlinked nuclear genes allow for multiple, independent datasets and, therefore, independent estimates of phylogenetic relationships. In contrast, the chloroplast and mitochondrial genomes provide single markers due to gene linkage [26], [29]. Furthermore, the higher rate of sequence evolution of low-copy nuclear genes [23], [25] may allow for greater phylogenetic resolution in clades containing slowly evolving or recently diverged taxa.

However, working with nuclear markers has its limitations. Successfully designing primers and amplifying target sequences can be quite difficult and labor-intensive steps such as cloning are often necessary. In order to confidently reconstruct species relationships it is of great importance to compare orthologous loci rather than paralogous copies [23]. Because most nuclear genes belong to multi-gene families with different lineages containing losses or duplications the search for orthology is a crucial limitation of working with nuclear genes [23], [30] and great care must be taken. Focusing on single- or low-copy nuclear genes, however, can alleviate this limitation.

In this study we compare results based on five chloroplast markers and 2 single-copy nuclear loci from the pentatricopeptide repeat (PPR) gene family. The phylogenetic utility of these nuclear loci for plant phylogenetic reconstruction has been previously demonstrated by Yuan *et al.* [31]. The PPR loci were found to have a single orthologue in both *Oryza sativa* and *Arabidopsis thaliana* and a rapid rate of evolution, useful at the intergeneric and interspecific levels [31]. Following empirical studies on Verbenaceae [32], recent studies have demonstrated the utility of these genes in plant phylogenetics [33], [34].

One of the strengths of using PPR loci is that orthology has been previously assessed [31]. Therefore, cloning is likely not necessary for these loci as primers were designed to specifically amplify orthologues. Additionally, because they are intronless, issues with highly polymorphic introns are avoided [31], [32].

In this study we evaluate the utility of two PPR loci to resolve evolutionary relationships within the Campanuloideae. Our results suggest that these markers, when considered separately and in combination with plastid data, can be informative tools for phylogenetic reconstruction and for the detection of putative hybridization events.

## Methods

### Taxon Sampling, Amplification, and Sequencing

Taxa spanning the Campanuloideae clade were included in this study (Fig. S1) to test the utility of two single-copy nuclear genes for reconstructing relationships across this large, taxonomically diverse group as well as resolving relationships between closely related species. *Cyphia elata* (Cyphioidae) and *Solenopsis minuta* (Lobelioideae) were used as outgroup taxa based on previous studies [6], [35], [36].

A number of chloroplast (*atpB*, *matK*, *petD, rbcL,* and *trnL-F*) and ITS sequences were taken from previously published works available from Genbank and additional taxa, including all PPR sequences, were amplified as described below (Fig. S1). Total genomic DNA was extracted from silica dried leaf tissue and herbarium specimens following a modified cetyltrimethyl ammonium bromide (CTAB) extraction protocol [37].

Nuclear (PPR) primers were designed after Yuan *et al*. [32]. We screened the primer pairs discussed in this study and found *AT1G09680* and *AT3G09060* to give clean results when PCR products were directly amplified, with very few polymorphic sites, suggesting a single copy of these loci within all tested individuals. Following Lu-Irving & Olmstead (2013), these will hereafter be referred to as PPR11 (*AT1G09680)* and PPR70 (*AT3G09060)*, from the order in which Yuan *et al*. [31] list them. We found that for the *PPR11* locus, 320F and 1590R primers were the most successful within the Campanuloideae (Table 1). For the *PPR70* locus, we used the 930F and 2080R primers. Both of these primer pairs were used for PCR amplification and sequencing. Chloroplast primer sequences used in this study are also shown in Table 1.

**Table 1:** Chloroplast and Nuclear Loci Used in this Study.

Locus	Primer Sequences (5′–3′)	Number ofTaxa	Total CharactersIncluded	VariableSites	ParsimonyInformative Sites	Model of MolecularEvolution
**atpB**	F: TATGAGAATCAATCCTACTACTTCT	119	1230	319	183	TPM1+G
	R: TCAGTACACAAACATTTAAGGTCAT					
**matK**	F: TTTCAGGARTATATTATGCACTTGCT	120	1315	679	438	GTR+I+G
	R: GCGAAATAGARGAAGCTCTGG					
**petD**	F: GCCGRMTTTATGTTAATGC	183	1228	596	420	SYM+G
	R: AATTTAGCYCTTAATACAGG					
**rbcL**	F: ATGTCACCACAAACAGARACTAAAGC	125	1179	328	198	TPM2
	R: GCAGTTATTGATAGACAGAAAAATCATGGT					
**trnL-F**	F: CGAAATCGGTAGACGCTACG	185	1024	428	245	GTR+I
	R: ATTTGAACTGGTGACACGAG					
**PPR11**	F: TTTGTTATGTTGATKTGGGTTTT	137	826	510	399	TPM3+I+G
	R: GCCAGAAATAATAGCCGTGTAAG					
**PPR70**	F: AGTGCTYTGATTCATGGGTTGTG	203	981	693	492	TVM+I+G
	R: ACAGCTCKRACAAGTATRTTCCA					
**Concatenated Plastid**		121	5973	2039	1252	GTR+I+G
**Concatenated PPR**		116	1807	1012	713	TIM3+I+G
**Concatenated Plastid+PPR**		121	7727	3047	1892	GTR+I+G

In order to further verify orthology, we screened eight taxa across the Campanuloideae for multiple copies of both PPR loci. Cloning followed the StratClone PCR Cloning Kit protocol (Stratagene) following the manufacturer’s instructions. Between two and eight colonies were picked, amplified, and sequenced using T7 and T3 primers. An initial phylogeny included directly sequenced PCR products as well as cloned sequences. Only a single sequence type of *PPR70* was found in all individuals. However, two distinct fragments were amplified using the *PPR11* primers for *Campanula pelviformis, C. glomerata,* and *C. tubulosa*. These two distinct sequence types differed greatly in nucleotide composition and size (approximately 300 bp and 850 bp). We were able to easily distinguish between the ‘large-copy’ and ‘small-copy’ by simple gel electrophoresis, suggesting cloning was unnecessary and paralogy was not an issue in this dataset if all amplified fragments were of appropriate length. Given a single band was visualized using gel electrophoresis for all subsequent taxa, we proceeded with direct sequence following amplification, without the need for cloning.

All new sequences were amplified in 50 μl PCR reactions containing: 1 μl DNA, 10 μl 5X buffer, 5 μl of 25 mM MgCl_2_, 10 μl Betain, 4 μl of 0.1 μM dNTPs, 5 μl of 5 μM primers, 1.25 units *Taq* polymerase (produced in the lab from *E. coli*), and water was added to bring to volume. Amplification reactions for nuclear loci were run on an automated thermal cycler under the following conditions: (1) initial denaturation was carried out at 95°C for 2 min; (2) five cycles of 95°C for 1 min, 53°C for 1 min, and 72°C for 2 min; (3) 32 cycles of 95°C for 1 min, 48°C for 1 min, and 72°C for 2 min; (4) a final elongation step at 72°C for 12 min. Plastid regions were amplified following Haberle *et al*. [6] and Borsch *et al*. [12].

Sequencing was carried out on an ABI Prism 3700 automated sequencer (Applied Biosystems). Sequences were inspected, assembled, and edited using Sequencher 4.9 (Gene Codes Corp., Ann Arbor, MI, USA). Initial alignments were carried out using Muscle [38] and manually adjusted in Se-Al v2.0 [39]. Polymorphic sites in heterozygotes were coded using standard IUPAC ambiguity codes. All sequences have been deposited in GenBank. All alignments have been included as supplementary files.

### Phylogenetic Analyses

JModelTest [40] was used to determine appropriate models of molecular evolution for all datasets using the Akaike Information Criterion (AIC) and comparing –*ln* likelihood scores. The best-fitting models for each dataset are given in Table 1.

All individual gene datasets were analyzed independently (*atpB*, *matK*, *rbcL*, *trnL-F, PPR11*, and *PPR70*) before we analyzed the concatenated chloroplast and PPR datasets. Because the individual datasets recovered largely congruent results, we combined the PPR and chloroplast loci, using the plastid dataset as a ‘guide’ and including only PPR accessions for which plastid data was also available. The combined plastid-PPR matrix included 124 ingroup taxa and 7727 characters. All datasets were analyzed using maximum likelihood and Bayesian Inference.

Maximum likelihood analyses were run in RAxML (version 7.0.4 [41]) using the most appropriate model for each dataset. The combined dataset was partitioned by gene. One thousand bootstrap replicates were generated to measure clade support. Bayesian analyses were conducted with MrBayes (version 3.1.2 [42], [43] with the following settings. The maximum likelihood model employed 6 substitution types (nst = 6), with rate variation across sites modeled using a gamma distribution, as well as a proportion of sites being invariant (rates = invgamma). Two Markov Chain Monte Carlo searches were run (Nruns = 2) with 4 chains each for 5000000 generations, with trees sampled every 1000 generations. We visually assessed convergence using AWTY [44].

Although a multi-species coalescent approach is likely to give more accurate results for multiple unlinked partitions when compared to analyses of concatenated datasets (e.g., [45]), our sampling of a single to a few individuals per species and only three independent loci is likely insufficient to accurately infer the species tree for Campanuloideae [46].

### Dating Analyses

Dating analyses were carried out with BEAST v1.7.4 [47] under an uncorrelated lognormal model. Twenty million generations were run logging parameters every 1000 generations. Tracer v.1.5 [47] was used to visualize log files, assess success of runs, and calculate “burn-in” for each analysis. Post burn-in trees were summarized with TreeAnnotator v.1.7.4 [47].

Although fossils for calibrating the Campanulaceae tree are limited, Campanuloideae fossil seeds are available. These fossils, identified as *Campanula* sp. and *Campanula paleopyramidalis*, date to the Miocene of the Nowy Sącz Basin in Poland [48], [49]. Geological and palynological studies have dated freshwater deposits of this formation to the Karpatian, approximately 17–16 MYA [50], [51], [52].

Following Cellinese *et al*. [5] we used the age of the well-determined *C. paleopyramidalis* fossil as a constraint for the most recent common ancestor of *C*. *pyramidalis* and *C*. *carpatica*. A lognormal prior distribution was applied to the fossil constraint with a mean of 5.0, stdev of 1.0, and offset of 16. This gave a minimum age constraint of 16 MYA for the node where the fossil was assigned, placing most of the prior probability on this younger age, but still allowing older ages for this constrained node. Placing this constraint on the most recent common ancestor of all *Campanula* species gave marginally younger ages, as expected (Crowl, unpublished data), without significantly changing our conclusions. Therefore, we restrict our discussion to the former analysis because we agree with the identification provided by Lancucka-Srodoniowa [48], [49].

We used two additional calibrations for the root of the tree based on a recent study [53], which estimated dates for a number of major angiosperm clades. Date ranges from the 95% highest posterior densities from this study were used to constrain the crown of the Campanulaceae (41–67 MYA) and the crown of the Campanuloideae (28–56 MYA). Normal distribution priors were placed on each of these nodes using the mean from each range reported in Bell *et al*. [53] as the mean for the prior distribution: 54 MYA for the Campanulaceae and 42 MYA for the Campanuloideae and a stdev of 5.0 for both.

## Results and Discussion

**Figure 1:**
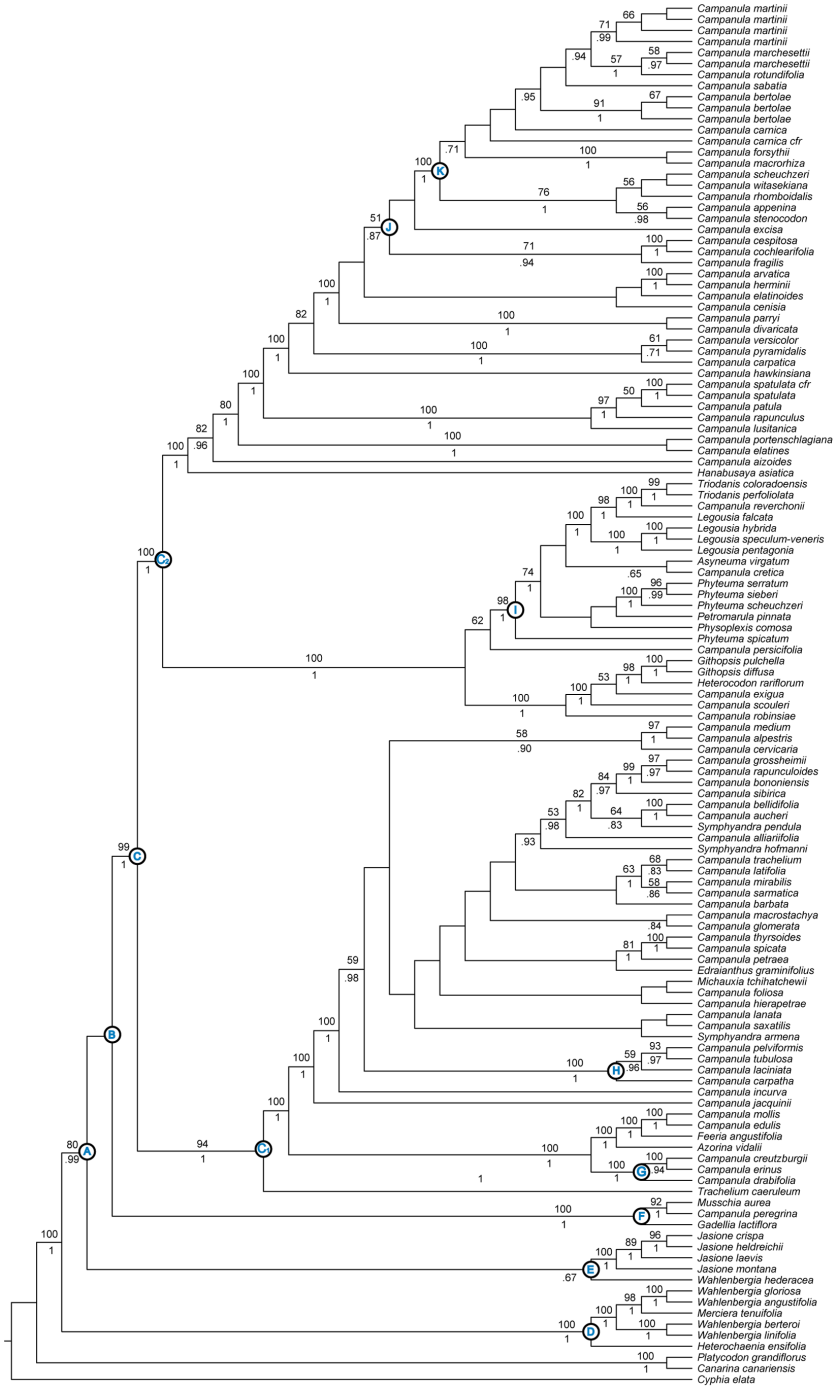
Plastid Phylogeny. Best tree from maximum likelihood analysis of combined plastid dataset: *atpB*, *matK*, *petD*, *rbcL*, and *trnL*-*F*. Numbers above branches are bootstrap values >50%. Numbers below branches indicate posterior probabilities >.70 from Bayesian analysis. Letters A-K refer to nodes and clades discussed in the text.

**Figure 2:**
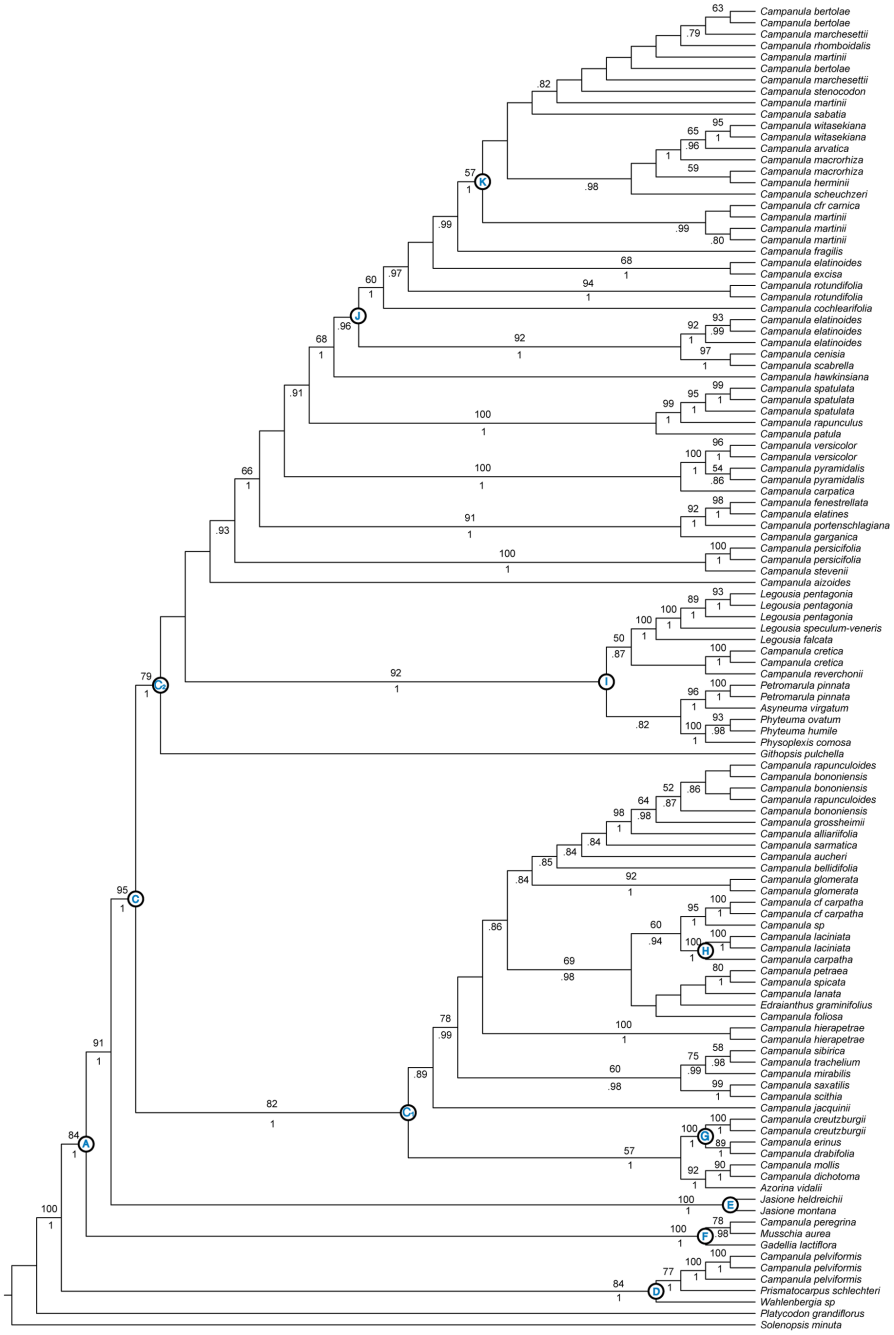
PPR Phylogeny. Best tree from maximum likelihood analysis of combined PPR dataset: *PPR11* and *PPR70*. Numbers above branches are bootstrap values >50%. Numbers below branches indicate posterior probabilities >.70 from Bayesian analysis. Letters refer to nodes and clades discussed in the text.

**Figure 3:**
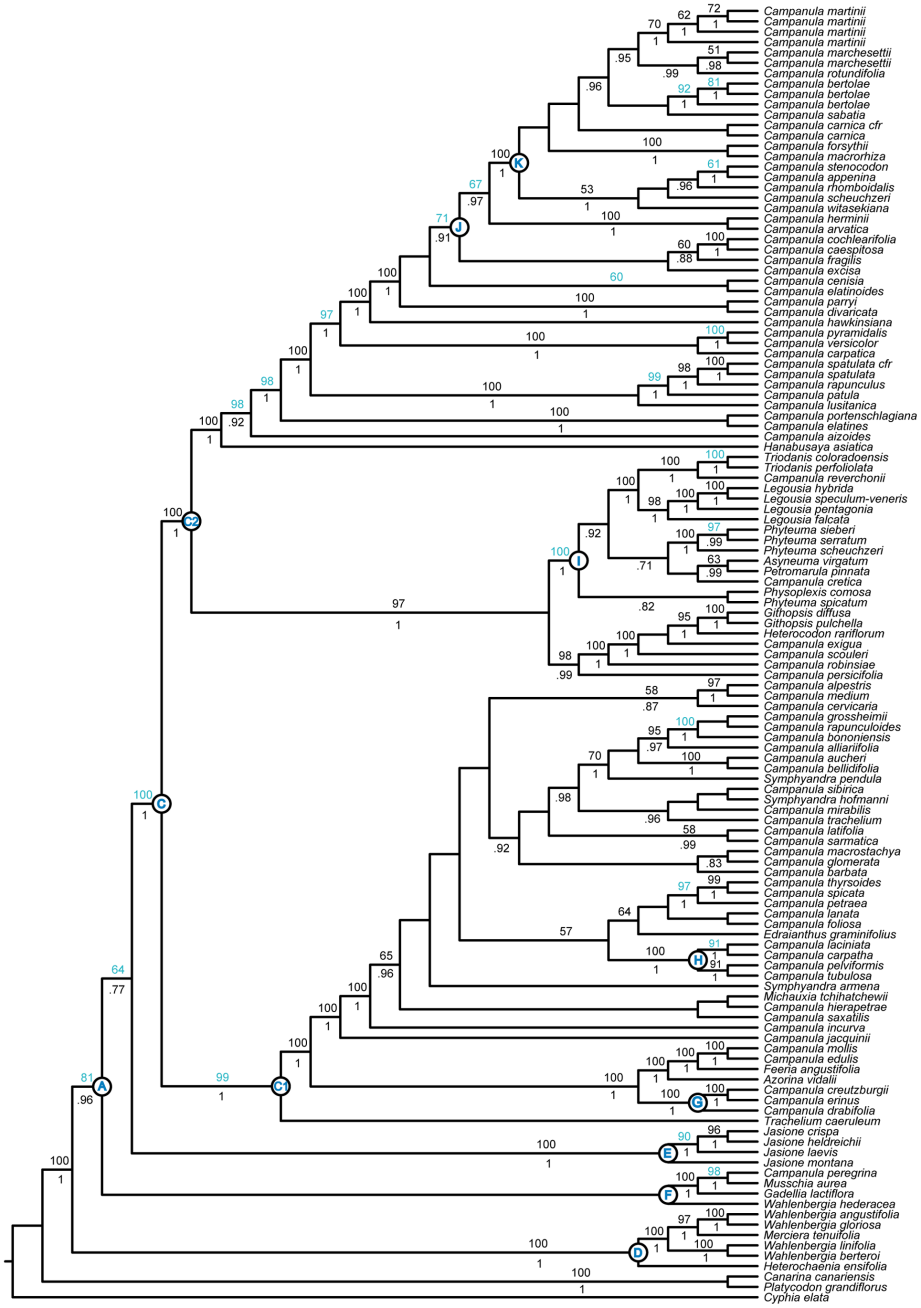
Combined Plastid and PPR Phylogeny. Best tree from maximum likelihood analysis of combined plastid-PPR dataset: *atpB*, *matK*, *petD*, *rbcL*, *trnL*-*F*, *PPR11*, and *PPR70*. Numbers above branches are bootstrap values >50%. Numbers below branches indicate posterior probabilities >.70 from Bayesian analysis. Letters refer to nodes and clades discussed in the text. Nodes for which bootstrap values are increased compared to the plastid-only analysis are highlighted in blue.

**Figure 4:**
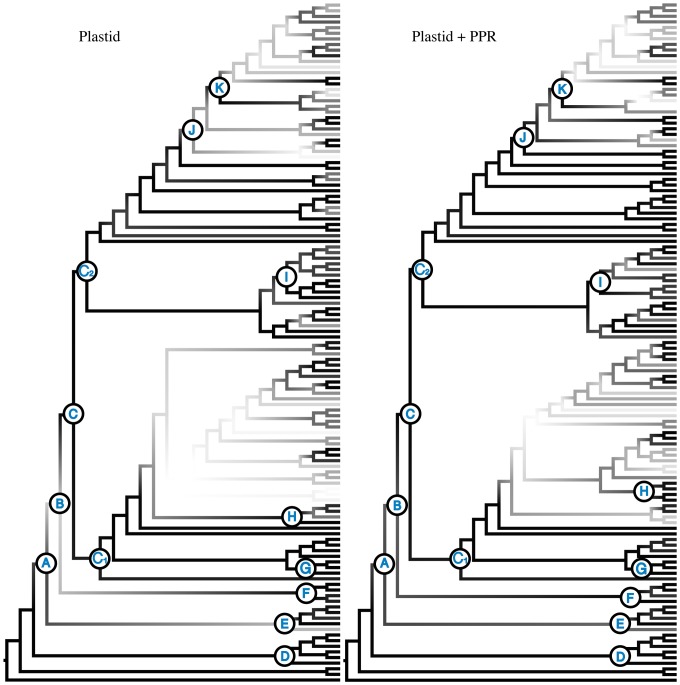
Comparison of Support for Plastid-only Tree and Combined Plastid-PPR Tree. Maximum-likelihood trees from the plastid dataset (a) and the combined plastid-PPR dataset (b) with taxon names removed. Branches are shaded relative to BS support with darker branches indicating higher support. Letters correspond to clade/node names in text and in Figure 1 and Figure 3. Support for many clades is increased with the inclusion of PPR loci while other areas of the tree remain poorly supported.

**Figure 5:**
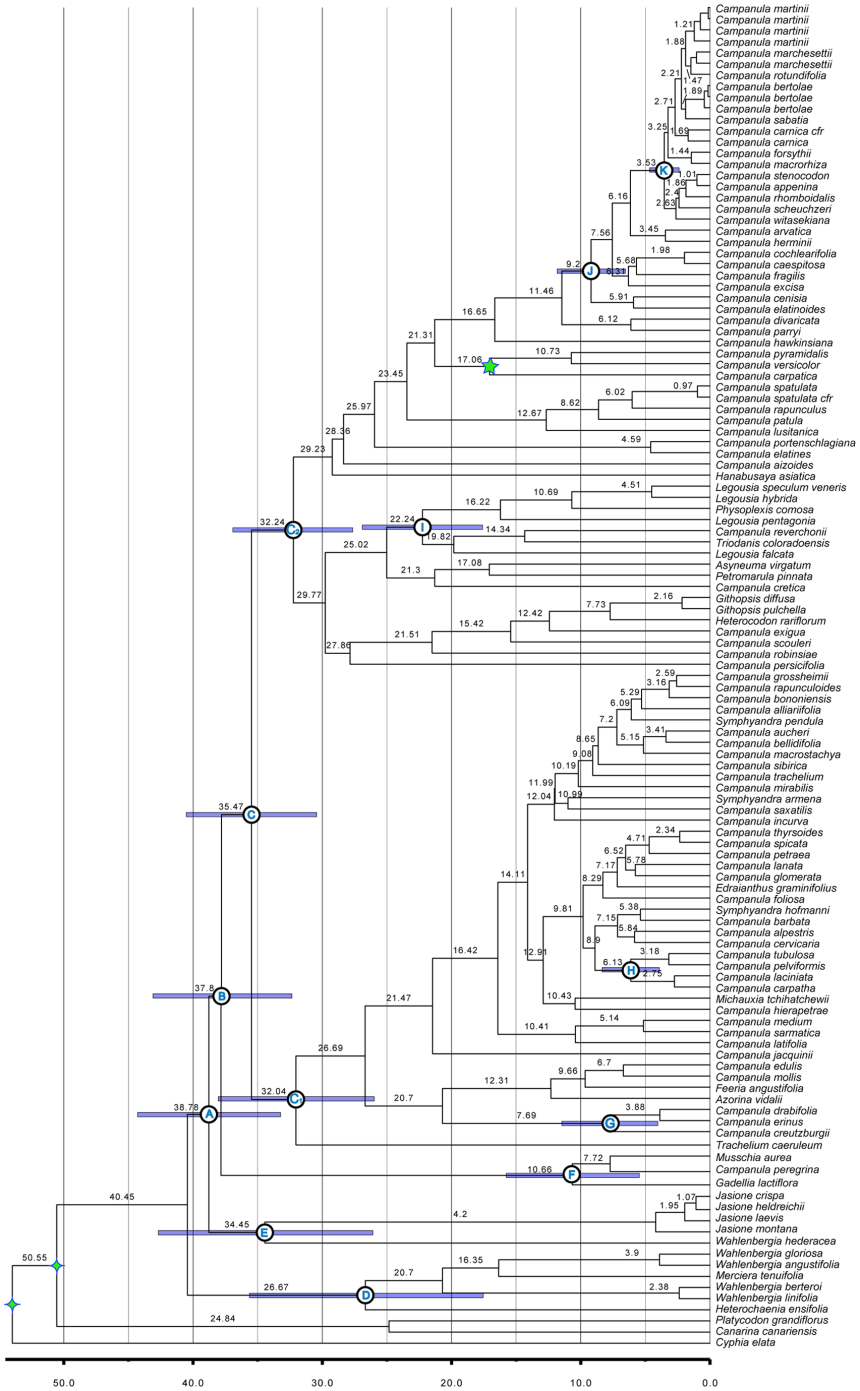
Divergence Time Estimates for Combined Plastid and PPR Tree. Chronogram from BEAST analysis of the combined plastid-PPR dataset. Scale bar is in millions of years before present. Green star indicates placement of fossil constraint. Green diamonds indicate age constraints obtained from a previous study [53]. Numbers above branches indicate mean age estimates for clades (in millions of years). Error bars around nodes correspond to 95% highest posterior distributions of divergence times for clades discussed in the text.

We generated 137 *PPR11* sequences and 203 *PPR70* sequences. The ITS matrix included 209 taxa. The final chloroplast matrices consisted of 119 *atpB* (Fig. S2), 120 *matK* (Fig. S3), 183 *petD* (Fig. S4), 125 *rbcL* (Fig. S5), and 185 *trnL-F* (Fig. S6) sequences (Table 1). Results from the plastid (Fig. 1), PPR (Fig. 2), and combined plastid-PPR (Fig. 3; Fig. 4) datasets are discussed below. Dating analyses of these three datasets gave similar results (see Figs. S12, S13, S14) and we restrict our discussion to the combined plastid-PPR dated phylogeny (Fig. 5).

Previous studies have attempted to utilize nuclear data to resolve relationships within the Campanuloideae by using the ribosomal internal transcribed spacer (ITS) region. As a way to further test the utility of PPR loci and directly compare results from the nuclear genome, we inferred relationships using ITS sequences obtained from GenBank (http://www.ncbi.nlm.nih.gov/genbank/). The ITS tree, despite a much larger taxon sampling than the PPR dataset, fails to provide significant resolution within the Campanuloideae clade (Fig. S19). Although we recovered congruence between these datasets, the sampling between them differs dramatically and we refrain from discussing these results in detail.

## Phylogenetic Resolution

### 

#### Plastid loci

Chloroplast loci have been the markers of choice for the majority of past phylogenetic studies on Campanulaceae. This is primarily due to the fact that universal primers are readily available and these regions can be rapidly amplified with relative ease. Furthermore, the high phylogenetic signal obtained at deep levels has made them attractive to studies aimed at gaining a general understanding of the Campanulaceae and resolving higher level relationships. Here we synthesize results from the five chloroplast-marker datasets and identify clades of interest for this study. Rather than discuss in detail each individual clade (which has been done in a recent study [8]), we simply highlight those that are relevant to our discussion on the utility and complications of the PPR markers. When appropriate, reference is made to the ‘Cam’ clade number of Mansion *et al*. [8] for each clade discussed in this study.

All individual markers gave largely congruent results (see Figs. S2–S6), which are generally in agreement with recent hypotheses [5], [6], [8], [11]. We, therefore, combined all chloroplast loci into a dataset that includes 124 ingroup taxa (Table 1). Figure 1 shows results from the Maximum Likelihood analysis of the combined plastid dataset. Bayesian analyses generated congruent results (see Figs. S9, S10, S11).

#### Clades A–C

The resolution and support along the backbone of the chloroplast phylogeny is largely consistent with past studies [5], [6], [8]. We found moderate support for the core Campanuloideae (*sensu* [12]; node A), which includes all *Campanula* species and close relatives. Node B is only weakly supported while we recovered strong support for node C and the two subclades, C_1_ and C_2_. Clade C contains the majority of Campanuloideae diversity and represents the split between two species-rich clades that include *Campanula* species and several segregate genera. The C_1_ and C_2_ clades, respectively, roughly correspond to the *Campanula s. str*. and *Rapunculus* groups defined by traditional taxonomic studies [3], [16], [54].

At the base of the Campanuloideae we found two clades that correspond to the Platycodonoideae, including *Platycodon* and *Canarina*, and the Wahlenbergioideae, including a polyphyletic *Wahlenbergia*. These results are also consistent with recent studies [6], [8], [16], [55].

#### Clade D

The Southern Hemisphere Clade D is strongly supported and represents the group that has been previously referred to as the Wahlenbergioideae [5], [6], [16]. The placement of this clade as sister to the core Campanuloideae is maximally supported and includes *Heterochaenia ensifolia* as sister to the polyphyletic *Wahlenbergia,* with the South African *Merciera tenuifolia* nested within the Southern Hemisphere *Wahlenbergia* species. *Wahlenbergia hederacea* falls outside of this clade and seems more closely related to *Jasione* (Clade E). This taxon is morphologically distinct from others in the genus and occurs in the northernmost range of *Wahlenbergia* [6]. The non-monophyly of *Wahlenbergia* has been consistently recovered in past studies [5], [6], [7], [8], [9], [55], [56].

#### Clade E

*Jasione* plus *Wahlenbergia hederacea* are found to be sister to the rest of the core Campanuloideae (Clade A). The placement of these taxa has been problematic in past studies. Analyses based on ITS by Eddie *et al*. [16] failed to resolve their relationship to other members of the Campanuloideae and indicated them as “transitional” taxa (transitional between “wahlenbergioids” and “campanuloids”). Although our combined chloroplast dataset recovers the core Campanuloideae with moderate support (node A) and strongly excludes *W. hederacea* from the Wahlenbergioideae clade, we fail to obtain support for the exact placement of *W. hederacea* or for this clade with respect to Clade F.

#### Clade F

(Cam01 in [8]). The next diverging group contains *Gadellia*, *Musschia*, and an Eastern Mediterranean *Campanula* species, *C. peregrina*. This clade is strongly supported but its placement within Clade A is unclear (node B). Similarly to Clade E, the exact placement of these “transitional” taxa [16] has been so far uncertain. Examining individual chloroplast gene trees reveals weak support and inconsistencies in the position of clades E and F, leading to overall weak support at node B (see Figs. S2–S6). Dating analyses (see combined plastid and PPR dataset discussion below**)** suggest rapid divergence of these two clades. Consequently, this dataset may contain too few synapomorphies to confidently place these taxa.

#### Clade G

**(**Cam14 in [8]). This complex, within the Campanula s. str. clade (C_1_), contains three morphologically similar species of annual *Campanulas* representing the *C. drabifolia* complex [57], sometimes referred to as the *Roucela* species complex (Crowl *et al*., in prep.) as taxa in this group are included in subgenus *Roucela* [1]. This maximally supported clade is composed of taxa restricted to the Mediterranean Basin - most narrowly endemic within the Aegean Archipelago. *C. drabifolia* (endemic to the mainland of Greece) is sister to a clade that includes the widespread *C. erinus* and the Cretan endemic *C. creutzburgii*. Our results are consistent with past studies [5], [6], [8], [55] that found the *C. drabifolia* complex as monophyletic and sister to a clade composed of western Mediterranean and North African taxa.

#### Clade H

This Cretan clade is recovered with high support. *Campanula pelviformis, C. carpatha*, *C. laciniata*, and *C. tubulosa* are all endemic to Crete and Karpathos islands except *C. laciniata*, which is also found in the Cyclades islands. This clade is likely the result of a single introduction into the Cretan area and one of the few examples of *in situ* diversification in the Cretan Campanuloideae [5].

#### Clade I

(Cam04 in [8]). This highly supported clade contains the paraphyletic *Legousia* (distributed primarily in southern Europe), and a North American clade containing *C*. *reverchonii* and *Triodanis* species. Our plastid results confirm the non-monophyly of *Legousia* with *L. falcata* sister to the North American clade [5], [6], [8], suggesting a single introduction into North America or possible hybridization (see PPR results and discussion). This North American-Mediterranean disjunction begs further study.

#### Clade J

(Cam12 in [8]). This poorly supported group is primarily composed of taxa distributed in central and southern Europe (Alps and Apennines). With the exception of Clade K and a couple species pairs (*C. cochleariifolia-cespitosa*, and *C. macrorhiza-forsythii*), very little resolution is obtained within Clade J with plastid markers.

#### Clade K

Clade K is composed primarily of taxa distributed in the Alps and adjacent areas with the exceptions of *C. forsythii*, endemic to Sardinia, and the widespread and taxonomically problematic *C. rotundifolia*, distributed throughout northern Europe and North America. Populations of *C. rotundifolia* show high phenotypic plasticity and the cytological studies of Kovanda [58] indicate at least two ploidy levels, diploid (2n  = 34) and tetraploid (2n  = 68). Although Clade J includes the majority of European alpine *Campanula* species, others are found within the C_1_ clade (e.g., *C. alpestris*), suggesting multiple introductions into the Alps.

### Pentatricopeptide Repeat (PPR) Loci

Both PPR loci (*PPR11* and *PPR70*) recovered relationships that are largely congruent with each other and with the chloroplast dataset (see Figs. S7 and S8). For example, the major split within the core Campanuloideae (Clade C: C_1_ and C_2_), the placement of the Wahlenbergioideae (Clade D), and other significant clades are recovered with both *PPR11* and *PPR70* datasets and are consistent with results based on chloroplast data. Therefore, we limit our discussion to the combined PPR (*PPR11* plus *PPR70*) dataset, which includes 111 ingroup taxa (Table 1).

The PPR dataset provides a well-resolved and highly supported backbone for the Campanuloideae phylogeny. Early diverging clades D-F are resolved with much higher support compared to the plastid tree (Fig. 2), though the placement of these clades is not always consistent (see below).

The Southern Hemisphere Wahlenbergioideae (Clade D) is maximally supported as sister to the core Campanuloideae (Clade A) and includes *Campanula pelviformis*. The *Musschia-Gadellia* clade (Clade F) is recovered as sister to the rest of the core Campanuloideae with moderate support (node A). This group predates the divergence of the *Jasione* clade (Clade E), which is strongly supported as sister to Clade C. Relationships along the backbone of the Campanuloideae have been problematic and this is the highest support obtained for the placement of these clades to date.

The placement of the maximally supported *C. drabifolia* complex (Clade G) sister to a clade containing western Mediterranean and North African taxa is consistent with plastid analyses.

The PPR dataset recovered a monophyletic *Legousia* sister to a clade containing a Cretan endemic (*C. cretica*) and a North American species (*C. reverchonii*) within Clade I. This is the first molecular study to suggest *Legousia* as monophyletic and, specifically, *L. falcata* as sister to the rest of the *Legousia* clade (Clade I; Fig. 2).

The phenomenon of incomplete lineage sorting is more prominent in recently diverged taxa [71]. Because of its diploid nature and biparental inheritance, the nuclear genome may have an effective population size four times larger than that of the chloroplast genome. The expected time to coalescence is therefore four times longer thereby increasing the probability of finding ancestral polymorphisms in taxa of recent origin when using nuclear loci [23], [29]. This poses a problem in the Campanuloideae as many taxa seem to be closely related and/or of recent origin (Fig. 5; [8], [55]). As a result, we conclude that lineage sorting is likely causing the lack of species monophyly in the PPR tree (Fig. 2). Given our sampling, however, the amount of non-monophyly inferred (one species with high support; discussed further in *Plastid–Nuclear Incongruence* section) using these markers is minimal.

Similar to the plastid tree, the PPR markers alone provided very little resolution within the C_2_ clade.

### Combined Plastid-PPR Loci and Dating Analyses

Because the results of the individual analyses were largely congruent, we chose to combine datasets in order to explore relationships of all included taxa and further explore the utility of the PPR loci. The combined plastid-PPR dataset included the same 121 taxa present in the chloroplast dataset.

This phylogeny is largely congruent with the results based on chloroplast data, likely because of a strong signal being contributed by the more numerous plastid markers. However, we found the addition of PPR loci to increase support at many nodes within the Campanuloideae phylogeny. Interestingly, the increase in support values spans from the backbone of the phylogeny (e.g., node A, node B, and node C) to the terminal lineages. In Figure 3 we highlight the 23 nodes for which BS support values increased compared to the plastid dataset. Figure 4 shows relative support for these two trees with darker branches indicating increasing support.

The combined dataset corroborates the placement of the *Jasione* and *Musschia* clades in the PPR tree (which contradicts the plastid results), though with only moderate support. We found the divergence of the *Musschia* clade (Clade F) to pre-date the divergence of the *Jasione* clade (Clade E). Although suggested before [8], [56], this is the strongest support to date for this relationship (Fig. 3). Dating analyses suggest a rapid divergence of these two clades in the Late Eocene (Fig. 5), providing a possible explanation for their uncertain placement.

As in the plastid dataset, *Wahlenbergia* is again found to be polyphyletic. However, the placement of *W. hederacea* is unsupported. A recent expanded phylogeny of *Wahlenbergia* supports the exclusion of *W. hederacea* from the Wahlenbergioideae clade [7], [9]. Although we were unable to test the monophyly of *Wahlenbergia* with both PPR loci, the individual *PPR11* analysis corroborates this relationship (see Fig. S7).

The combined plastid-PPR dataset places *Campanula pelviformis* in the Cretan Clade H rather than Clade D as in the PPR tree (Fig. 2), consistent with the chloroplast results. Again, this is likely the result of five chloroplast markers contributing more phylogenetic signal than the two PPR loci. Dating analyses support the hypothesis that Clade H may have been the result of an *in situ* radiation in the Cretan area [5] with the stem of this clade dating to approximately 9 million years old and diversification of the crown clade estimated at approximately 6 million years ago (Fig. 5).

Analysis of the combined plastid-PPR dataset inferred *Legousia* to be monophyletic with strong support (Fig. 3, Clade I). Three North American species (*Triodanis* and *C. reverchonii*) form a clade sister to the *Legousia* clade. We are currently investigating the relationship of *Legousia* with North American taxa at a finer scale using complete taxon sampling and both plastid and nuclear data (Crowl *et al*., in prep).

The combined plastid-PPR dataset recovered increased support for Clade J, with *Campanula cenisia* and *C. elatinoides* in a sister relationship and sister to this clade. A clade containing *C. excisa*, *C. fragilis*, *C. cochleariifolia*, and *C. caespitosa* is found to be sister to the rest of this clade with *C. herminii* and *C. arvatica* diverging next and strongly supported as sisters. The overall resolution and support within Clade I is increased when PPR loci are combined with the plastid matrix (Fig. 3). However, many relationships within Clade K could not be resolved with statistical confidence even when nuclear loci were included (Fig. 4). This is likely due to the recent origin of this clade (Fig. 5) and possibly a rapid radiation into the Alps.

Our results infer non-monophyly of Alpine and Caucasian taxa, indicating multiple introductions into these areas, likely during the Pleistocene glaciation. Dating analyses indicate all of these taxa diversified prior to the major glaciation of northern and central Europe during the Pleistocene and, therefore, may have originated in different areas. Both of these areas acted as refugia for many taxa during times of unfavorable climatic conditions [59], [60], [61] and it is likely that many Campanuloideae taxa followed this or a similar pattern.

Of the six species that were found to be non-monophyletic in the PPR tree, three of these (*Campanula martinii*, *C. marchesettii,* and *C. bertolae*) were found to be monophyletic in the combined analyses (Clade K; Fig. 3). We were unable to assess the monophyly of the remaining three taxa because sequence data for multiple accessions was not available.

Dating analyses indicate the Campanuloideae to be approximately 50 million years old and the diversification of the core Campanuloideae (Clade A) beginning approximately 38 million years ago. Much of the diversification of this group, however, occurred within the last 5–10 million years (Fig. 5), potentially complicating phylogenetic reconstruction.

### Plastid–nuclear Incongruence

As discussed above, results from individual datasets gave largely congruent results. However, we did discover discordant patterns between plastid and nuclear loci. Topological contradictions between the two datasets are discussed below.

Lack of allelic monophyly within nuclear gene datasets and incongruence between nuclear and plastid datasets may be caused by lineage sorting, gene flow, or gene duplication leading to paralogous copies [62], [63], [64], [65]. Because no paralogy issues were detected within the PPR dataset, the incongruences found here are likely due to incomplete lineage sorting or hybridization.

Six species are found to be non-monophyletic in the PPR tree, although support is low. These include *C. bononiensis* and *C. rapunculoides* in Clade C_1_, and *C. elatinoides,* the recently described *C. martinii* [66], *C. bertolae,* and *C. marchesetii* in Clade J. *C*. *elatinoides* is the only species for which the non-monophyly is well supported. Much of Campanuloideae diversity has occurred very recently (within the last 10 MYA; Fig. 5; [8]) and, consequently, rapid divergence events may have hindered lineage sorting. Therefore, the lack of allelic monophyly for these six taxa could be the result of incomplete lineage sorting. Alternatively, recent hybridization events may also explain these results. Many of the taxa found to be non-monophyletic have overlapping or adjacent distributions in Italy and across the Alps, suggesting past or recent hybridization events may be likely. However, further investigation into phenology and other potential sources of pre- or post-zygotic reproductive isolation between these sympatric species is required to fully understand the patterns we have found here.

The placement of Clade E and Clade F are inconsistent between datasets with the divergence of Clade E pre-dating the divergence of Clade F in the plastid tree (Fig. 1). This relationship is reversed in the nuclear tree, and with higher support. This pattern is again recovered in analyses of the combined plastid plus nuclear dataset. Dating analyses indicate these two clades diverged within approximately one million years of each other (Fig. 5). The disagreement in the placement of these clades between plastid and nuclear markers – and even between studies using plastid data – is likely to be the result of an ancient, rapid radiation (Fig. 5).

Comparing PPR and chloroplast trees reveals a possible ancient hybridization event. While the chloroplast dataset places *Campanula pelviformis* in a clade containing other Cretan taxa (Clade H in Fig. 1), the PPR dataset places this taxon near the base of the Campanuloideae in Clade D, with *Prismatocarpus* and *Wahlenbergia*, two Southern Hemisphere taxa. Multiple accessions of *C*. *pelviformis* were included in our analyses, to verify the unusual placement of this taxon. This result is quite surprising, however, and we cannot discount the possibility that this pattern is due to retention of ancient paralogues in PPR11 or some other misleading phenomenon that begs in-depth investigation into this taxon.

We also uncover hybridization as a possible force in the history of *Legousia*. Our results based on the chloroplast dataset (Fig. 1) are in agreement with other studies that have consistently found *Legousia* to be non-monophyletic. Analyses based on the PPR dataset, however, infer this group as monophyletic with strong support (Clade I; Fig. 2), a result not previously recovered. While the plastid tree indicates *Campanula reverchonii* closely related to *L. falcata* (Fig. 1), the nuclear tree recovered this taxon in a clade sister to a monophyletic *Legousia* (Clade I; Fig. 2). The fact that the non-monophyly occurs in the plastid tree while monophyly is recovered with the nuclear dataset suggests that incomplete lineage sorting is unlikely (see discussion below). Two possible scenarios could explain the paraphyletic *Legousia* inferred with plastid data. First, this could be the result of hybridization. Alternatively, this may be the result of a long distance dispersal event from within the *Legousia* clade to North America. In this case, the paraphyly inferred is simply due to insufficient time for plastid loci to reach reciprocal monophyly. We are currently investigating this issue in more detail (Crowl *et al*., in prep.).

While we can suggest general patterns of hybridization in Campanuloideae, it is difficult to infer specific hybridization events because of incomplete taxon sampling (the Campanuloideae include over 1,000 extant species, of which we have sampled approximately 11% here). However, the increased phylogenetic resolution afforded by these nuclear loci will make them useful in future studies aimed at disentangling relationships within clades of closely related taxa and/or species complexes where complete or near-complete sampling is possible (Crowl *et al*., in prep). Furthermore, although we present likely scenarios regarding hybridization events, it is difficult to distinguish between the processes of incomplete lineage sorting and hybridization in causing discordance between gene trees. This is a very active area of research and recent studies have suggested methods in which species trees are employed to distinguish between these two processes (eg. [67], [68], [69], [70]). We leave this for future studies, where complete taxon sampling of specific clades is possible.

## Conclusions

This study represents the first inclusion of low-copy nuclear markers for phylogenetic reconstruction in Campanuloideae. The PPR loci included here present a powerful tool for Campanulaceae phylogenetics as they provide independent estimations of relationships, allowing researchers to uncover hybridization events and, when used in combination with plastid data, yield increased resolution at both deep and shallow phylogenetic levels. These loci were easy to sequence, required no cloning, and the sequence alignments were straightforward across a large taxonomic breadth.

Our analyses recovered known relationships - often with increased statistical support - and suggested relationships not previously recovered. For example, we resolved the placement of two early diverging groups, the *Jasione* clade and *Musschia*-*Gadellia* clade, with increased confidence. Because of the putative ancient, rapid diversification that seems to have occurred (Fig. 5), rapidly evolving markers such as the PPR loci are necessary to capture this event and resolve the placement of such clades.

Consistent with past studies, we find further evidence for the non-monophyly of *Wahlenbergia*. Although we failed to find support for the precise placement of *W. hederacea*, the non-monophyly of this group has now been corroborated by studies employing plastid, nrITS, and low-copy nuclear data.

PPR loci analyzed alone and in combination with plastid data also recovered relationships not previously suggested. Our results indicate that *Legousia* is, in fact, a monophyletic group, as expected when considering morphology, a result missed when only considering data from the chloroplast genome. The paraphyly inferred by chloroplast analyses may be due to past hybridization, or the result of a single, long-distance dispersal event from within the *Legousia* clade to North America. In the case of the latter, the non-monophyly consistently recovered with plastid data is the result of an insufficient amount of time passing for these loci to reach reciprocal monophyly. This result highlights the importance of using multiple, independent loci when inferring phylogenetic relationships and assessing taxon monophyly.

In order to obtain a comprehensive understanding of the evolutionary history of the Campanulaceae, it is apparent that numerous independent, rapidly evolving loci will be needed. Although many relationships still remain unresolved, likely due to the recent origin and rapid diversification of many *Campanula* species (Fig. 5), the inclusion of the PPR loci presented here bring us one step closer to inferring a species level phylogeny of this diverse clade of angiosperms. These markers may be of great use especially in studies aimed at clades in which complete or near complete sampling is possible, allowing for the discovery of past hybridization events, an aspect of a taxon’s evolutionary history not captured when only organellar markers are considered.

## Supporting Information

Figure S1 **Accession table.** Genbank accession numbers for DNA sequences used in this study.
(XLSX)

Figure S2 **ML** ***atpB*** **tree.** Individual *atpB* gene tree inferred with maximum likelihood.
(TIF)

Figure S3 **ML** ***matK*** **tree.** Individual *matK* gene tree inferred with maximum likelihood.
(TIF)

Figure S4 **ML** ***petD*** **tree.** Individual *petD* gene tree inferred with maximum likelihood.
(TIF)

Figure S5 **ML** ***rbcL*** **tree.** Individual *rbcL* gene tree inferred with maximum likelihood.
(TIF)

Figure S6 **ML** ***trnL-F*** **tree.** Individual *trnL-F* gene tree inferred with maximum likelihood.
(TIF)

Figure S7 **ML** ***PPR11*** **tree.** Individual *PPR11* gene tree inferred with maximum likelihood.
(TIF)

Figure S8 **ML** ***PPR70*** **tree.** Individual *PPR70* gene tree inferred with maximum likelihood.
(TIF)

Figure S9 **Bayesian plastid tree.** Combined plastid tree inferred with MrBayes.
(TIF)

Figure S10 Bayesian PPR tree. PPR tree inferred with MrBayes.
(TIF)

Figure S11 **Bayesian plastid plus PPR tree.** Combined plastid plus PPR tree inferred with MrBayes.
(TIF)

Figure S12 **Plastid chronogram.** Chronogram from BEAST analysis of plastid dataset.
(TIF)

Figure S13 **PPR chronogram.** Chronogram from BEAST analysis of PPR dataset.
(TIF)

Figure S14 **Plastid plus PPR chronogram with confidence intervals.** Chronogram from BEAST analysis of plastid plus PPR loci showing 95% HPD.
(TIF)

Figure S15 **Plastid alignment.**
(TXT)

Figure S16 **PPR alignment.**
(TXT)

Figure S17 **Plastid plus PPR alignment.**
(TXT)

Figure S18 **ITS alignment.**
(TXT)

Figure S19 **ML ITS tree.** ITS gene tree inferred with maximum likelihood.
(TIF)

Figure S20 **Plastid ML tree.** Newick tree format.
(TXT)

Figure S21 **PPR ML tree.** Newick tree format.
(TXT)

Figure S22 **Plastid plus PPR ML tree.** Newick tree format.
(TXT)
