# Supplementary figures and images for: Phylogeny of Campanuloideae (Campanulaceae) with Emphasis on the Utility of Nuclear Pentatricopeptide Repeat (PPR) Genes
Source: PLoS One. 2014 Apr 9;9(4):e94199. doi: 10.1371/journal.pone.0094199 (PMC3981779; doi:10.1371/journal.pone.0094199)

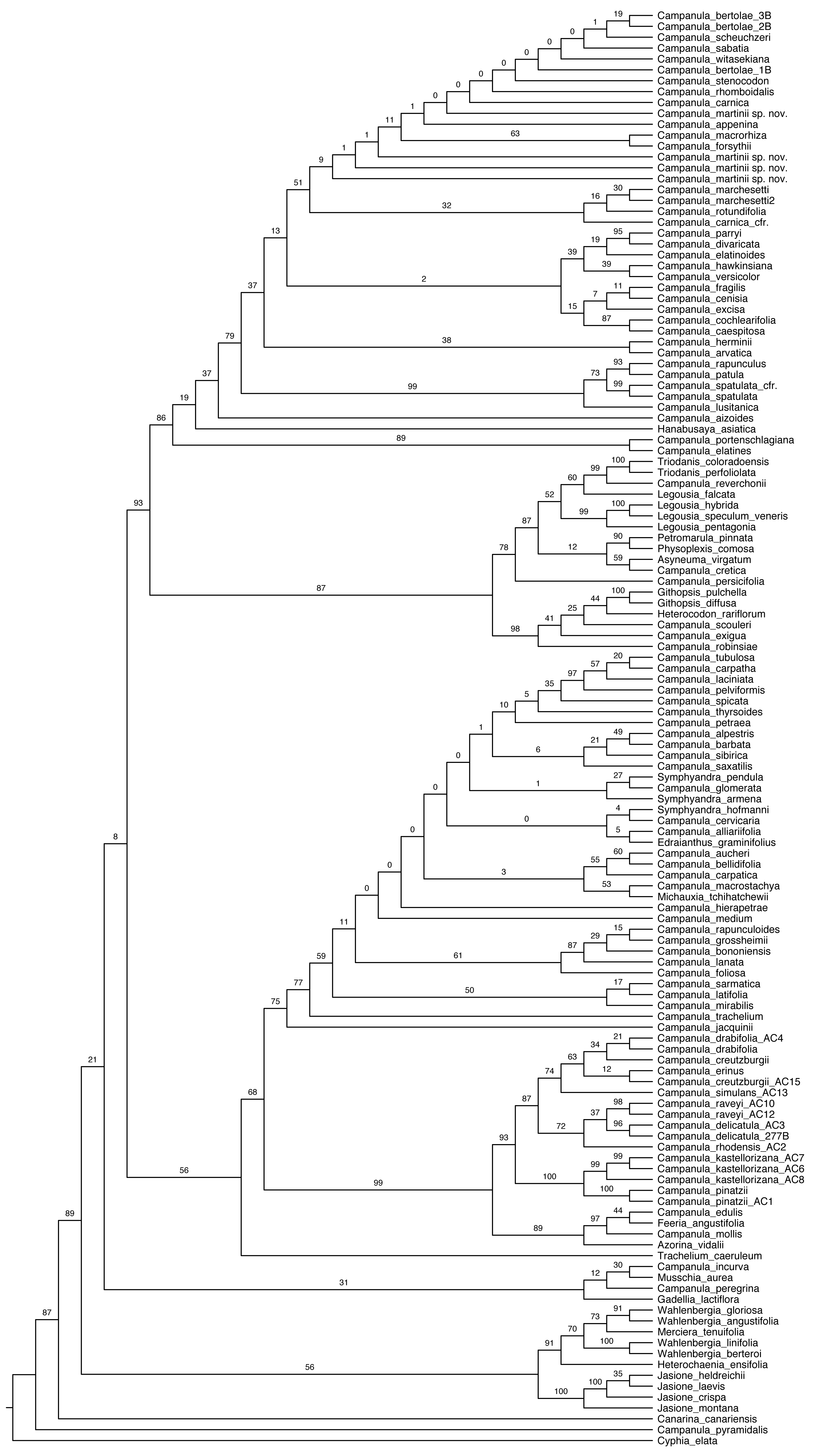

Supplement: Figure S2 — ML atpB tree. Individual atpB gene tree inferred with maximum likelihood. (TIF) [file pone.0094199.s002.tif]

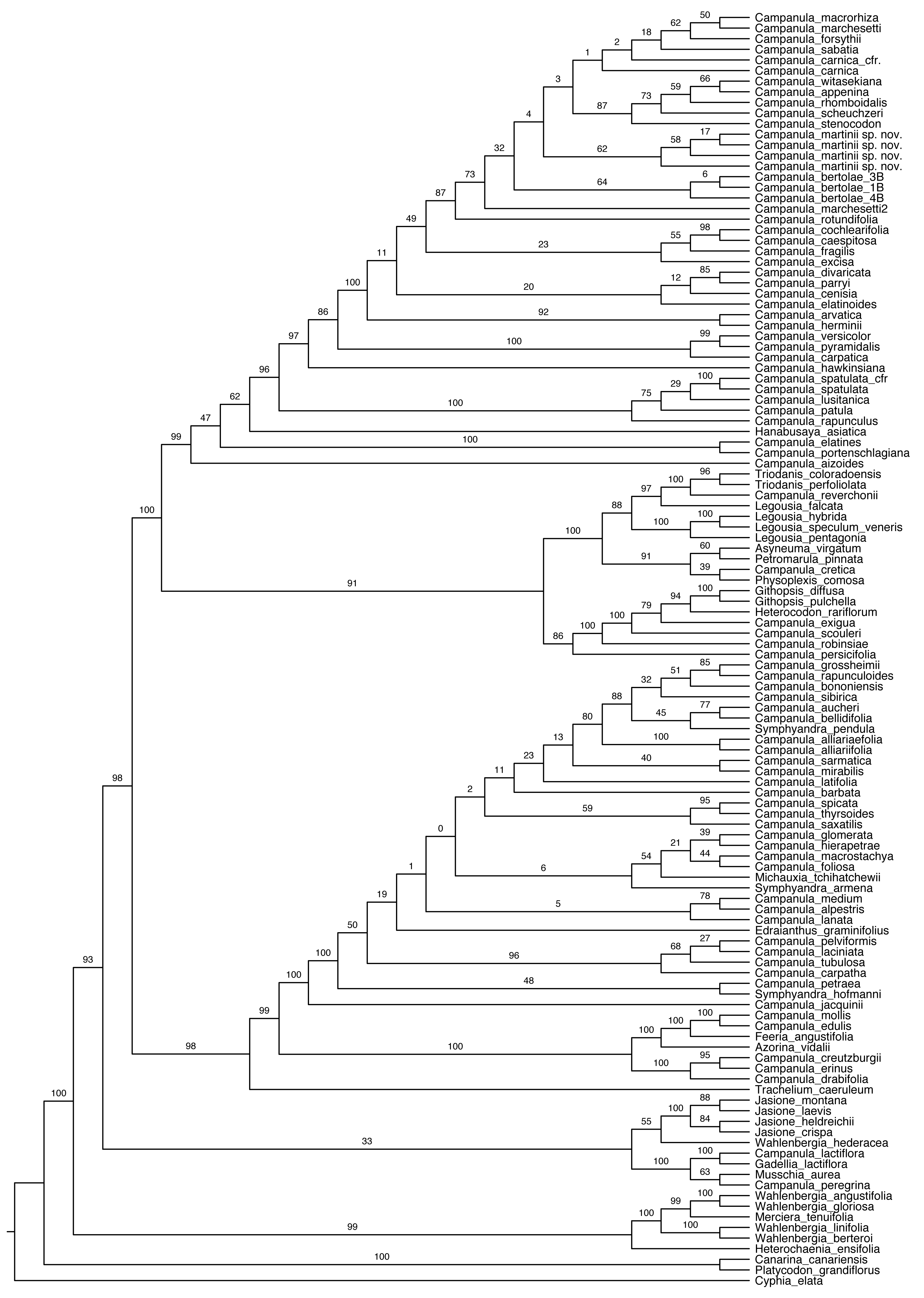

Supplement: Figure S3 — ML matK tree. Individual matK gene tree inferred with maximum likelihood. (TIF) [file pone.0094199.s003.tif]

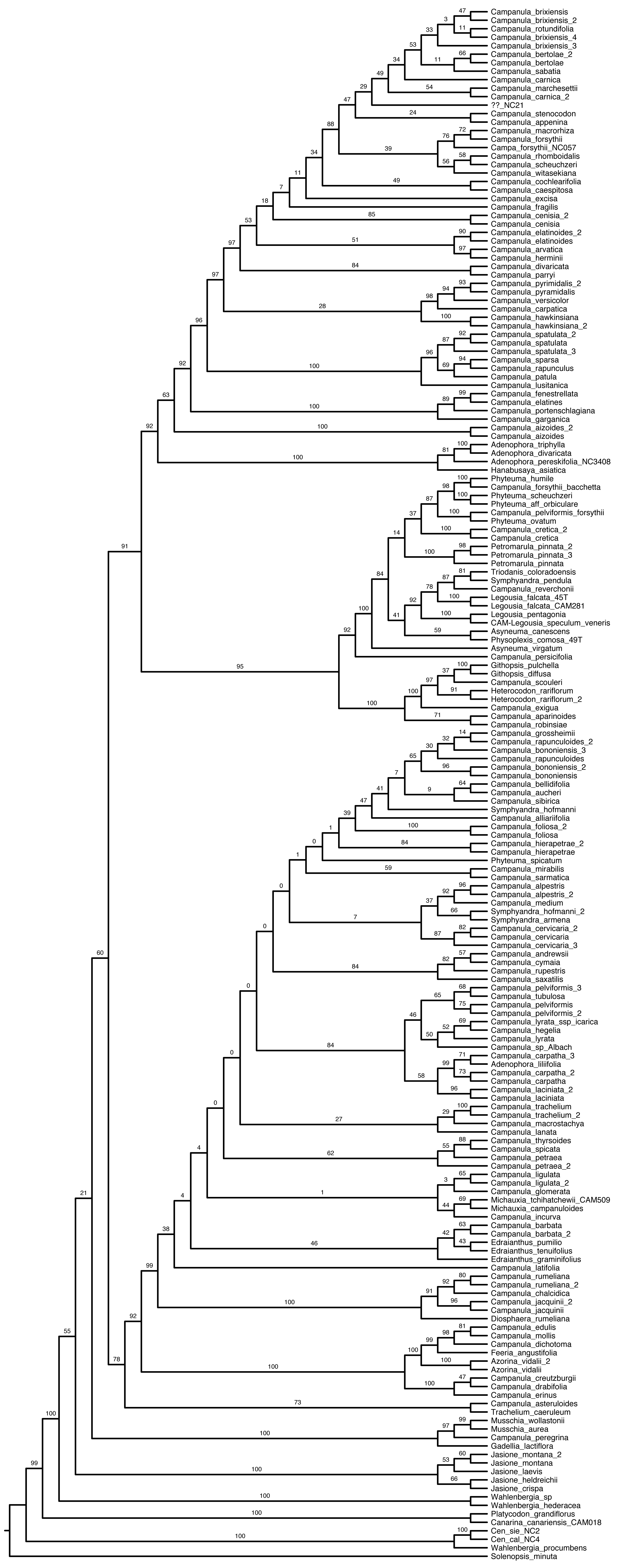

Supplement: Figure S4 — ML petD tree. Individual petD gene tree inferred with maximum likelihood. (TIF) [file pone.0094199.s004.tif]

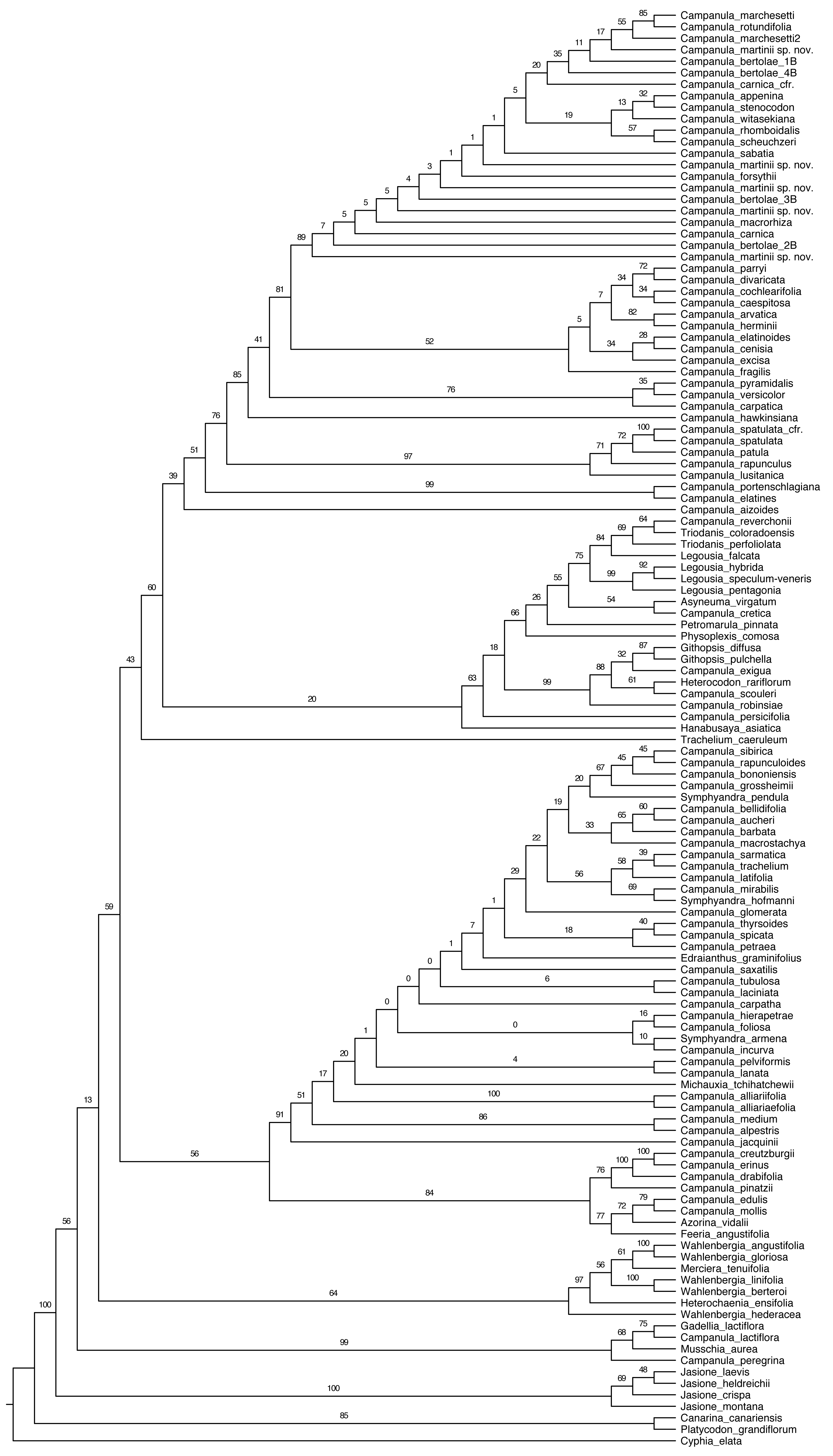

Supplement: Figure S5 — ML rbcL tree. Individual rbcL gene tree inferred with maximum likelihood. (TIF) [file pone.0094199.s005.tif]

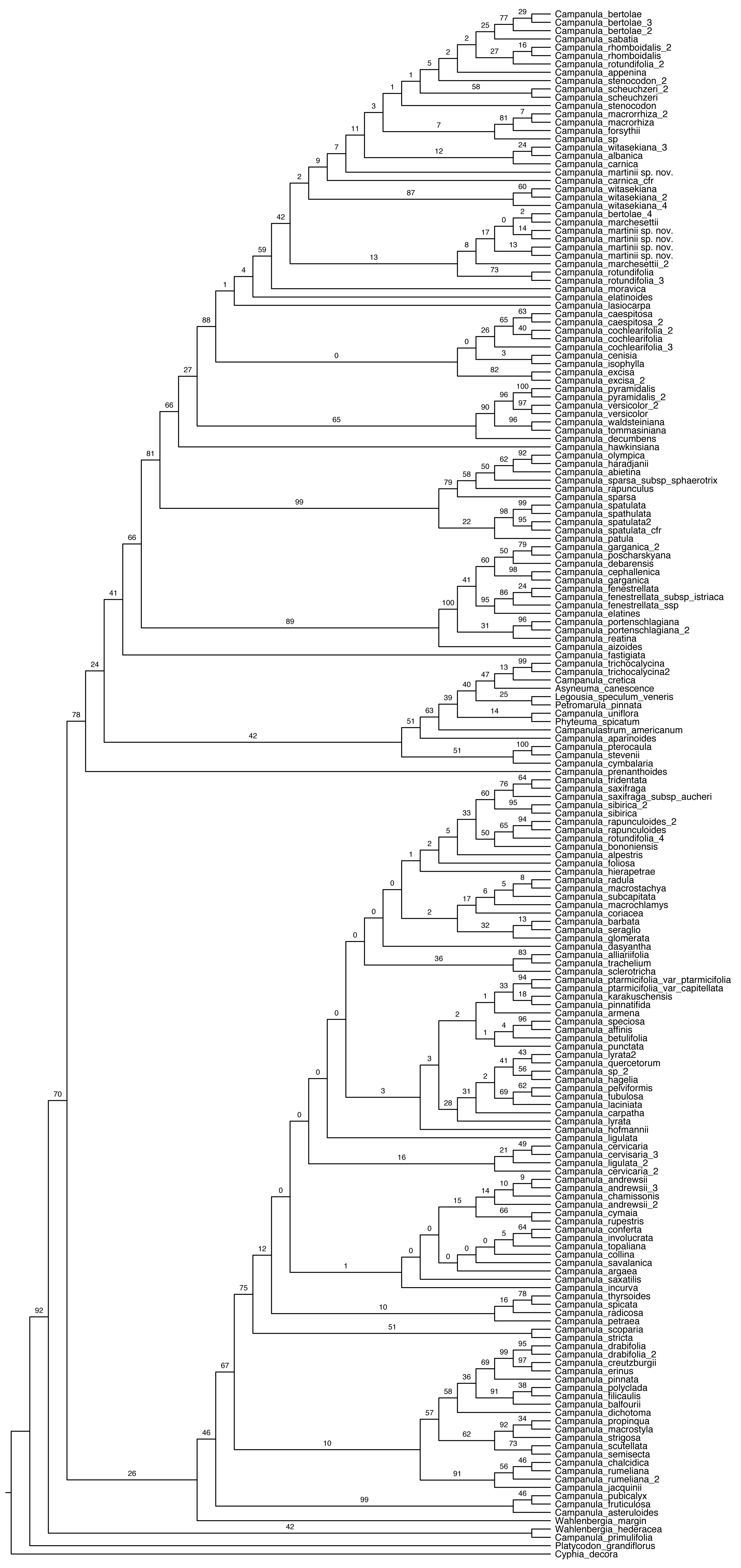

Supplement: Figure S6 — ML trnL-F tree. Individual trnL-F gene tree inferred with maximum likelihood. (TIF) [file pone.0094199.s006.tif]

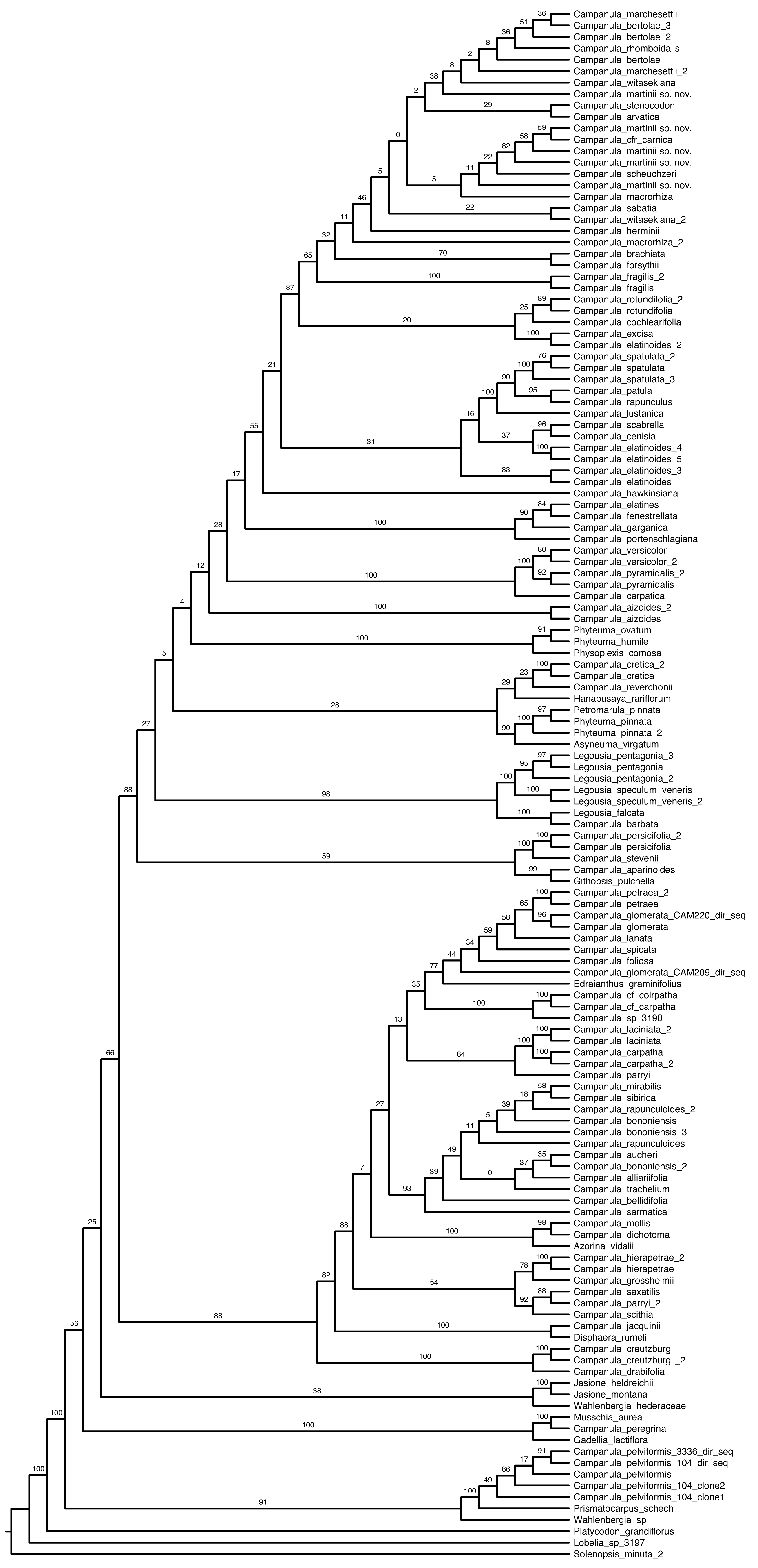

Supplement: Figure S7 — ML PPR11 tree. Individual PPR11 gene tree inferred with maximum likelihood. (TIF) [file pone.0094199.s007.tif]

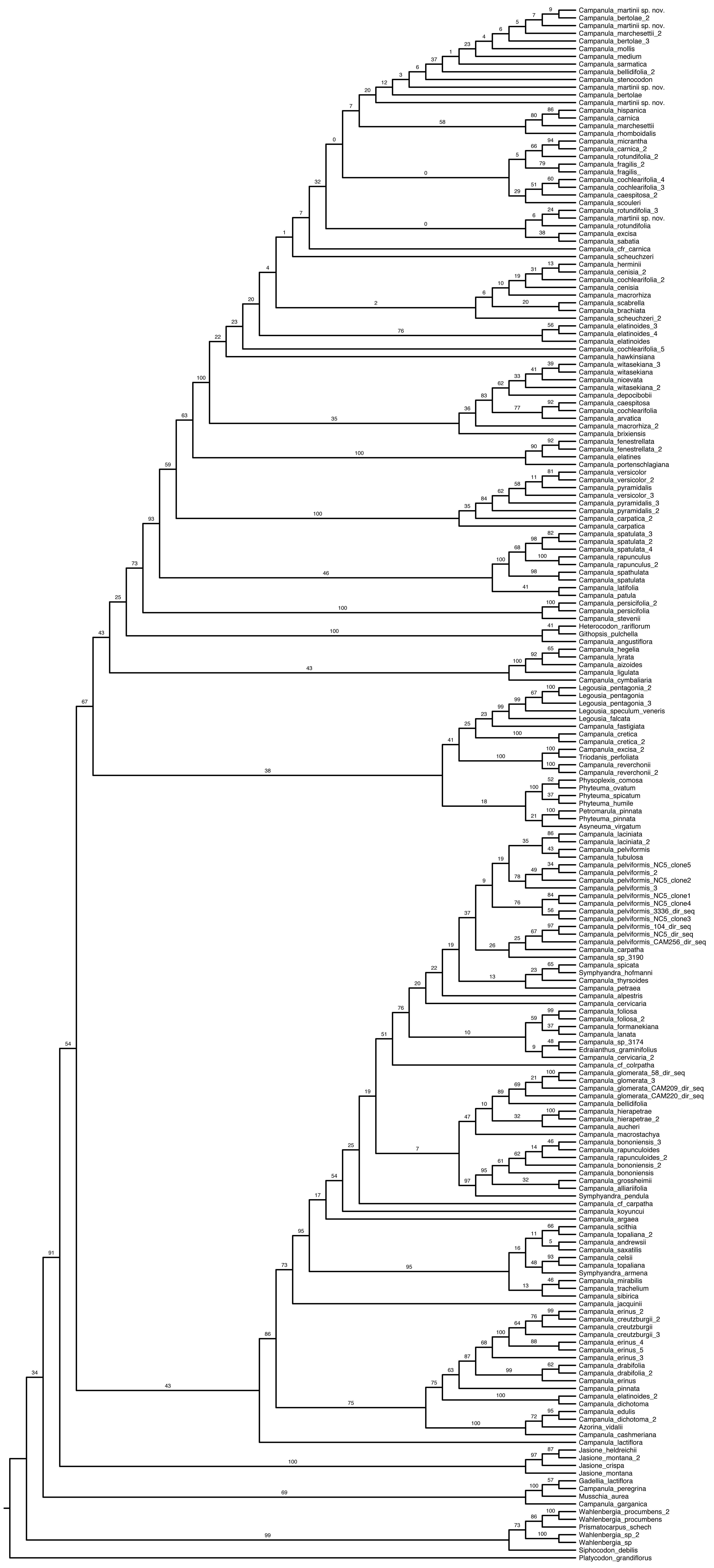

Supplement: Figure S8 — ML PPR70 tree. Individual PPR70 gene tree inferred with maximum likelihood. (TIF) [file pone.0094199.s008.tif]

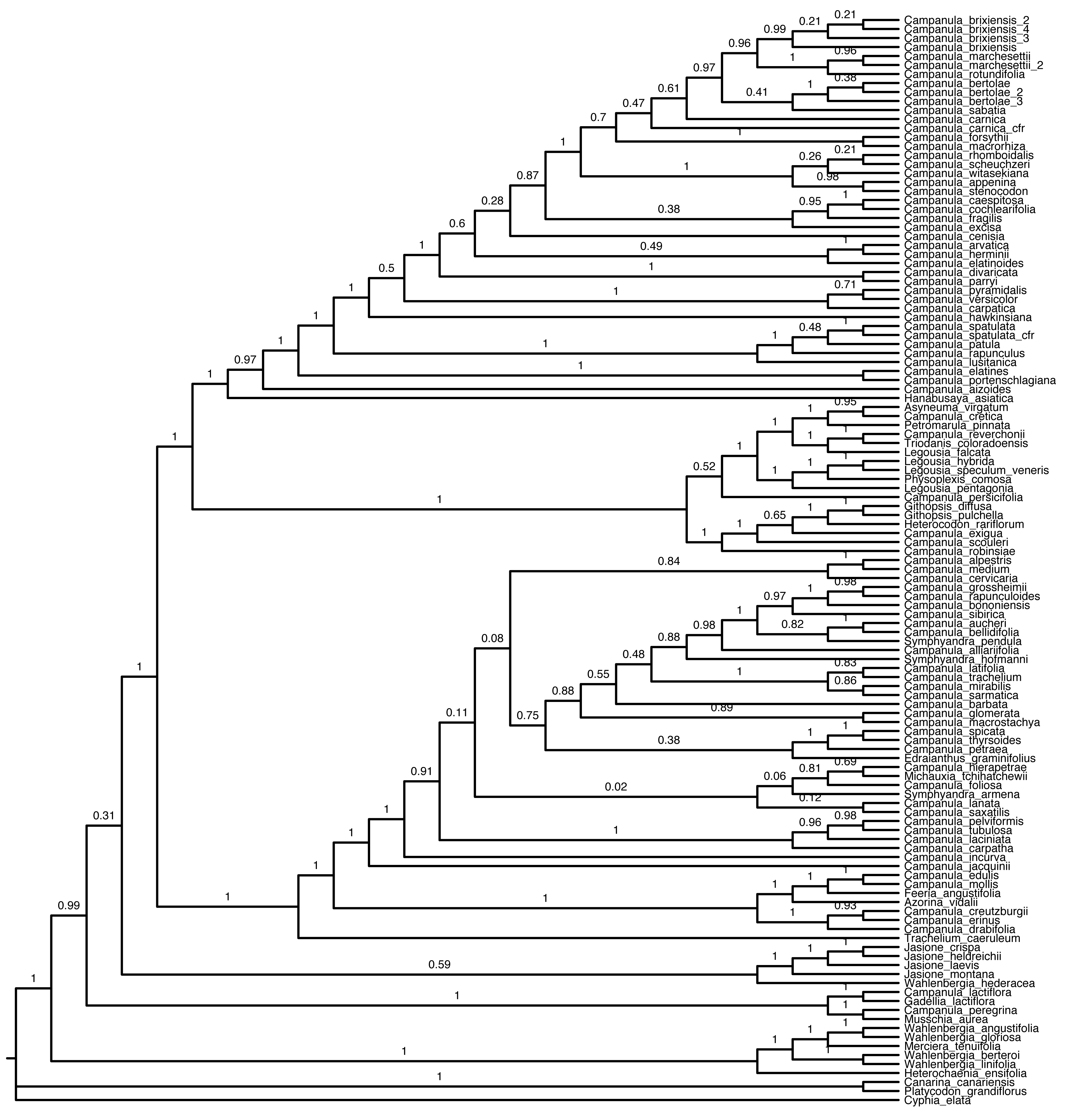

Supplement: Figure S9 — Bayesian plastid tree. Combined plastid tree inferred with MrBayes. (TIF) [file pone.0094199.s009.tif]

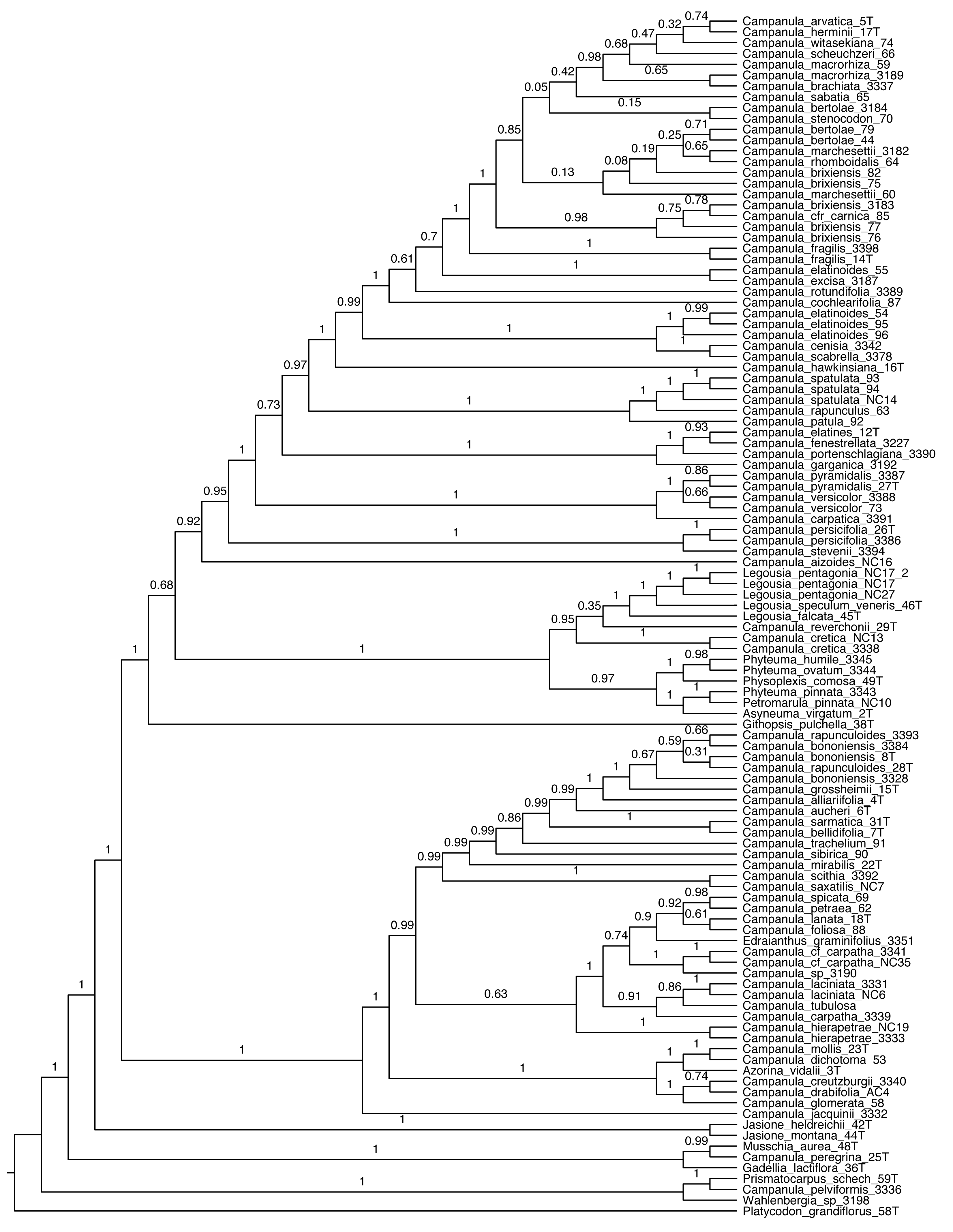

Supplement: Figure S10 — Bayesian PPR tree. PPR tree inferred with MrBayes. (TIF) [file pone.0094199.s010.tif]

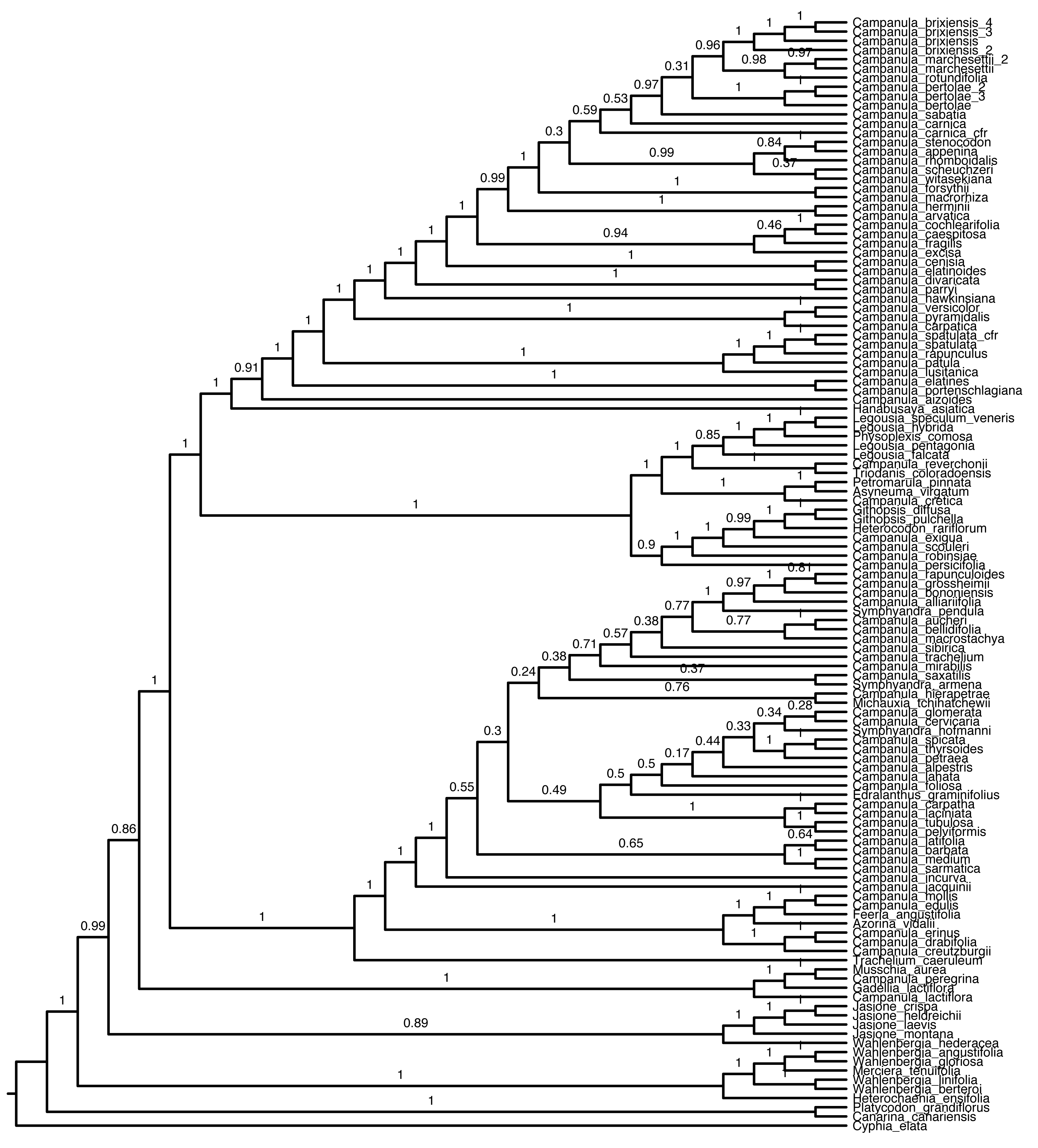

Supplement: Figure S11 — Bayesian plastid plus PPR tree. Combined plastid plus PPR tree inferred with MrBayes. (TIF) [file pone.0094199.s011.tif]

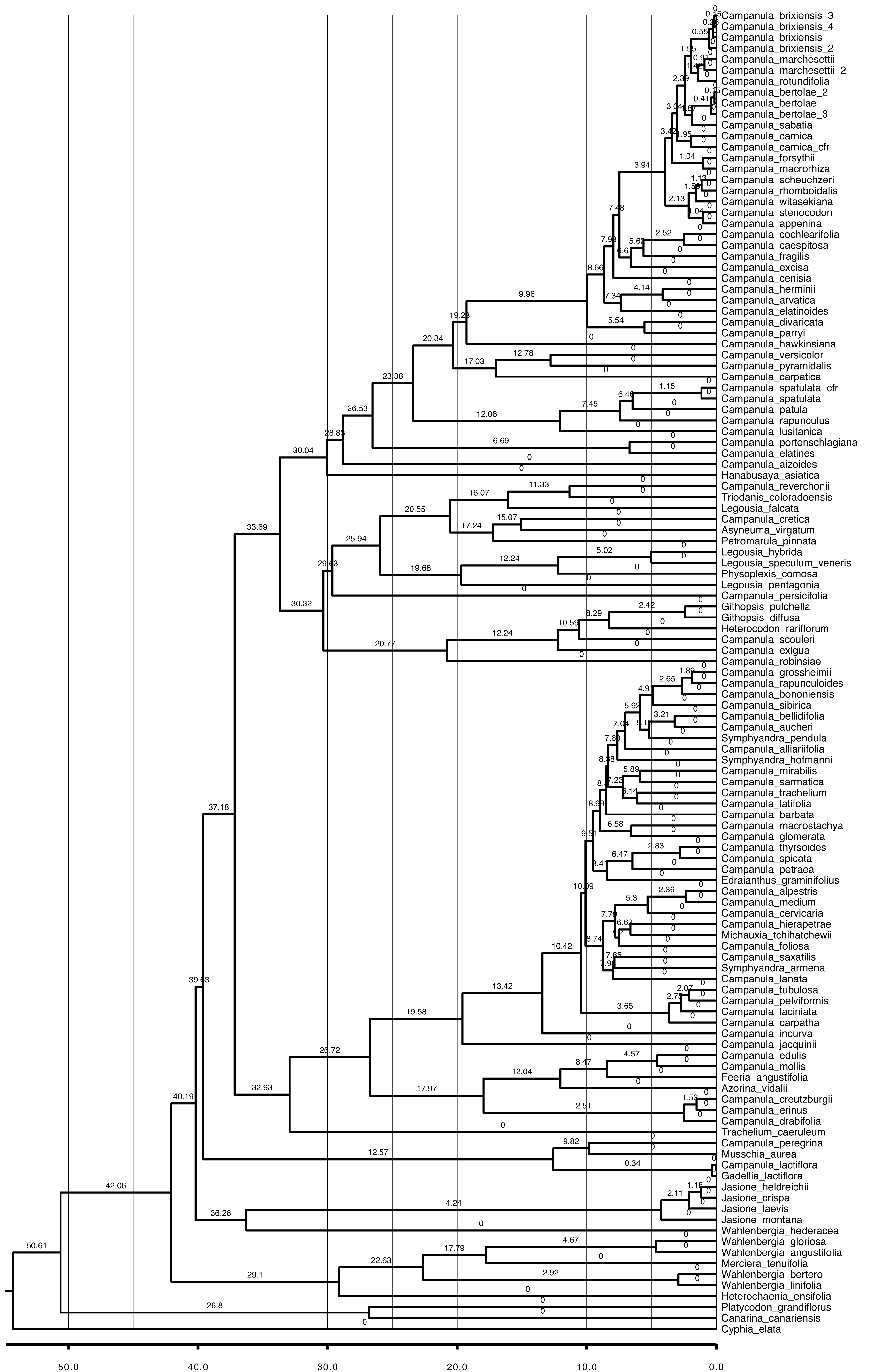

Supplement: Figure S12 — Plastid chronogram. Chronogram from BEAST analysis of plastid dataset. (TIF) [file pone.0094199.s012.tif]

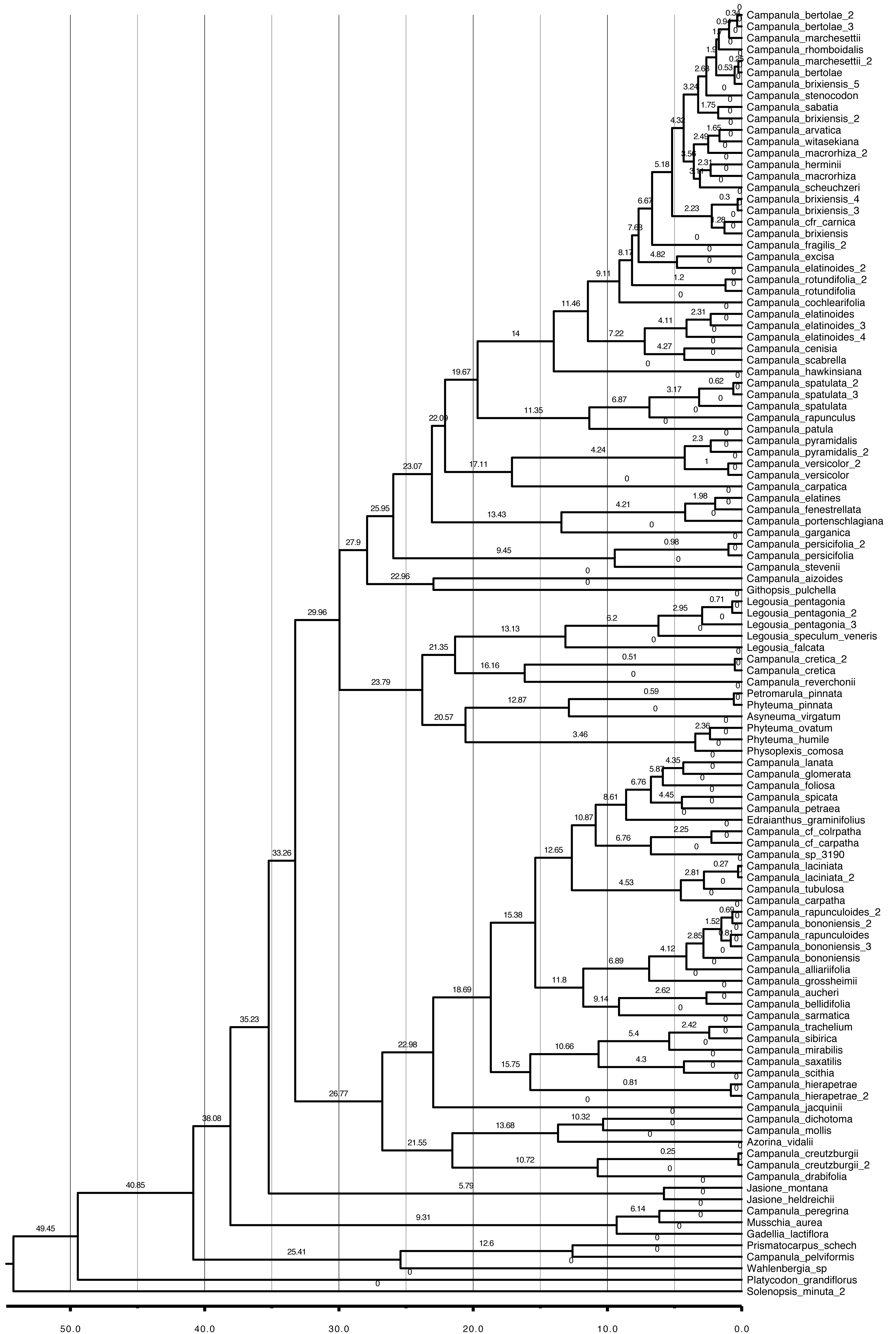

Supplement: Figure S13 — PPR chronogram. Chronogram from BEAST analysis of PPR dataset. (TIF) [file pone.0094199.s013.tif]

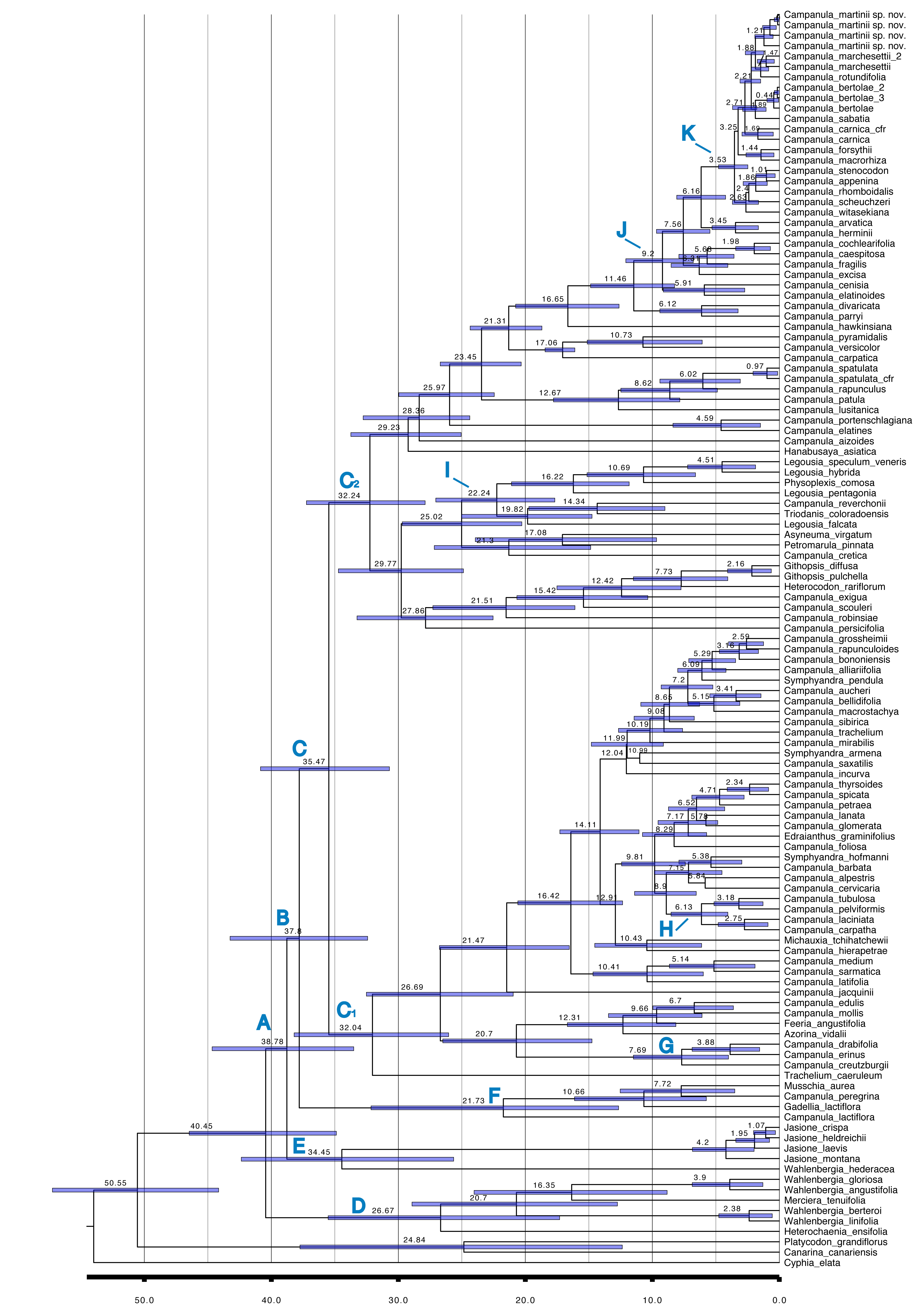

Supplement: Figure S14 — Plastid plus PPR chronogram with confidence intervals. Chronogram from BEAST analysis of plastid plus PPR loci showing 95% HPD. (TIF) [file pone.0094199.s014.tif]

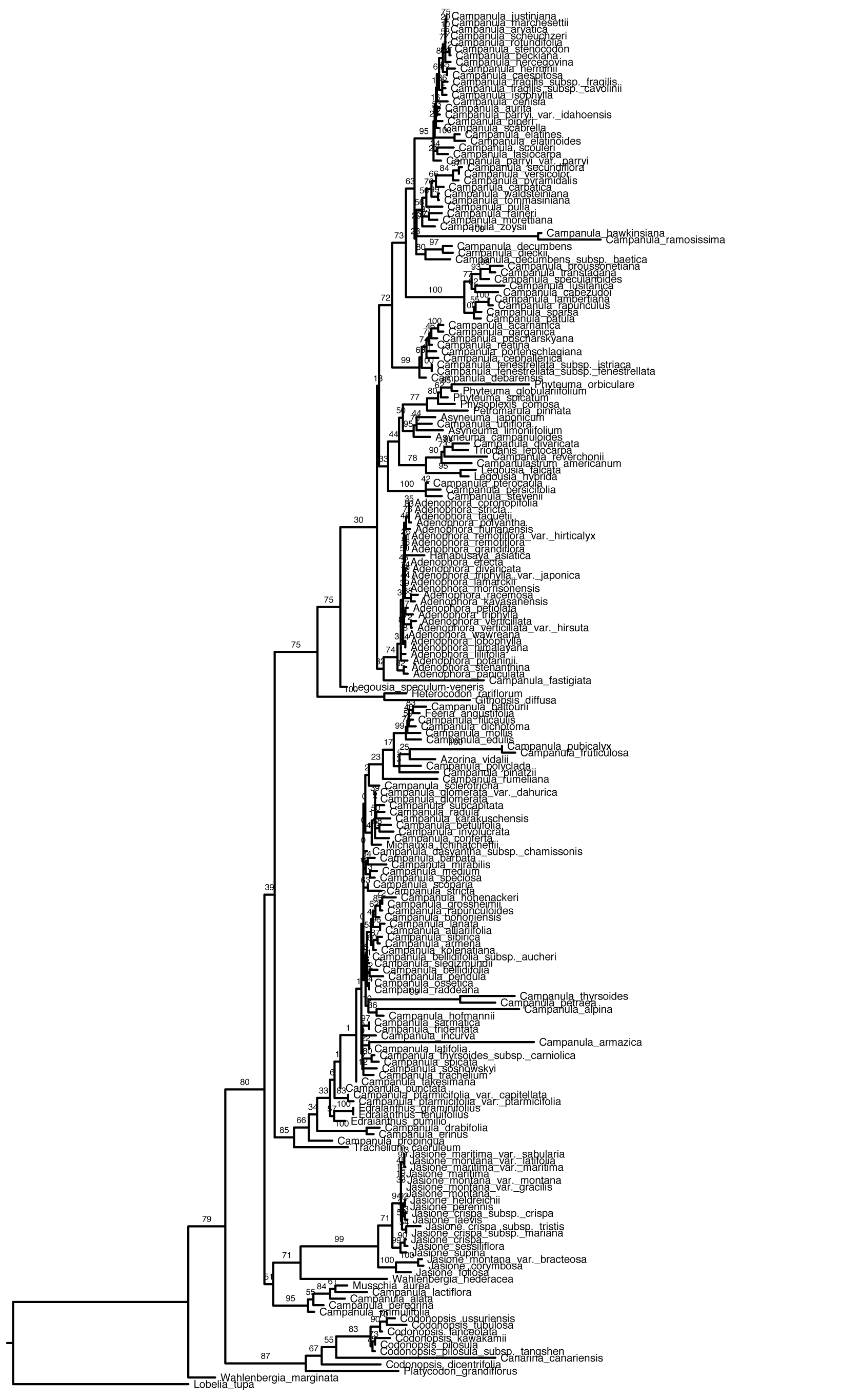

Supplement: Figure S19 — ML ITS tree. ITS gene tree inferred with maximum likelihood. (TIF) [file pone.0094199.s019.tif]
